# A novel aminotransferase gene and its regulator acquired in *Saccharomyces* by a horizontal gene transfer event

**DOI:** 10.1186/s12915-023-01566-6

**Published:** 2023-05-08

**Authors:** Sebastián M. Tapia, Laura G. Macías, Roberto Pérez-Torrado, Noemi Daroqui, Paloma Manzanares, Amparo Querol, Eladio Barrio

**Affiliations:** 1 Instituto de Agroquímica y Tecnología de los Alimentos, IATA-CSIC, Paterna, Spain; 2 Departament de Genètica, Universitat de València, Valencia, Spain

**Keywords:** *Saccharomyces*, HGT, Dialkylglycine decarboxylase, *DGD1*, AIB

## Abstract

**Background:**

Horizontal gene transfer (HGT) is an evolutionary mechanism of adaptive importance, which has been deeply studied in wine *S. cerevisiae* strains, where those acquired genes conferred improved traits related to both transport and metabolism of the nutrients present in the grape must. However, little is known about HGT events that occurred in wild *Saccharomyces* yeasts and how they determine their phenotypes.

**Results:**

Through a comparative genomic approach among *Saccharomyces* species, we detected a subtelomeric segment present in the *S. uvarum*, *S. kudriavzevii*, and *S. eubayanus* species, belonging to the first species to diverge in the *Saccharomyces* genus, but absent in the other *Saccharomyces* species. The segment contains three genes, two of which were characterized, named *DGD1* and *DGD2*. *DGD1* encodes dialkylglicine decarboxylase, whose specific substrate is the non-proteinogenic amino acid 2-aminoisobutyric acid (AIB), a rare amino acid present in some antimicrobial peptides of fungal origin. *DGD2* encodes putative zinc finger transcription factor, which is essential to induce the AIB-dependent expression of *DGD1*. Phylogenetic analysis showed that *DGD1* and *DGD2* are closely related to two adjacent genes present in *Zygosaccharomyces*.

**Conclusions:**

The presented results show evidence of an early HGT event conferring new traits to the ancestor of the *Saccharomyces* genus that could be lost in the evolutionary more recent *Saccharomyces* species, perhaps due to loss of function during the colonization of new habitats.

**Supplementary Information:**

The online version contains supplementary material available at 10.1186/s12915-023-01566-6.

## Background

Yeasts of the *Saccharomyces* genus have been used for different industrial processes such as winemaking, brewing, and bakery. Many studies have aimed to dissect the molecular foundations that underline the traits of biotechnological interest such as those related to nutrient consumption. Among the mechanisms responsible for the acquisition of new functions, the horizontal (or lateral) gene transfer (HGT or LGT) has gained more attention as an important evolutionary process involved in fungi and yeast adaptation to specific environments [1]. HGT is the acquisition from a distantly related species of xenologous genes, which provide either improved or novel biological functions that might confer a competitive advantage during environmental selective pressures [2].

In recent years, the vast availability of genome sequences of different yeast species and strains allowed the identification of more genes likely acquired through HGT [3, 4]. They are normally detected through phylogeny inference, where the topology of a candidate gene contradicts the established species’ phylogeny [1, 2]. In *S. cerevisiae*, most reported HGT events encode putative proteins related to metabolic processes such as the metabolism and transport of carbon and nitrogen sources [3, 5]. However, few studies have experimentally validated their function and physiological role. A well-reported prokaryote-to-eukaryote gene transfer in *S. cerevisiae* is the gene *URA1*, encoding a dihydroorotate dehydrogenase, which was acquired from a lactic acid bacterium to allow the anaerobic biosynthesis of orotate. It is believed this was an important evolutionary step for the adaption of *S. cerevisiae* to anaerobic environments [5]. In addition, eukaryote-to-eukaryote gene transfers have also been described in wine *S. cerevisiae* isolates which conferred an important advantage to growth in fermentative environments. In particular, the large genomic segments named regions B and C were acquired from *Zygosaccharomyces bailii* and *Torulaspora microellipsoides*, respectively [3, 6]. Region C contains the *FOT1-2* genes, encoding oligopeptide permeases, and the gene *FSY1*, codifying for a high-affinity fructose/H + symporter, which conferred the ability to uptake oligopeptides and the high fructose amounts present in the grape must, respectively [6, 7]. Nevertheless, there is still little experimental evidence about which HGT events have occurred in natural niches among wild *Saccharomyces* isolates.

Through a genomic comparative approach, we found a small 7-kb subtelomeric segment, containing three putative genes related to nitrogen metabolism, present in the species *S. uvarum*,* S. kudriavzevii*, and *S. eubayanus*, but absent in the remaining *Saccharomyces* species. In the present study, we report the characterization of two of these genes, one named *DGD1* encoding a yeast dialkylglycine decarboxylase, and the second *DGD2* codifying its putative regulator*.*

**Fig. 1:**
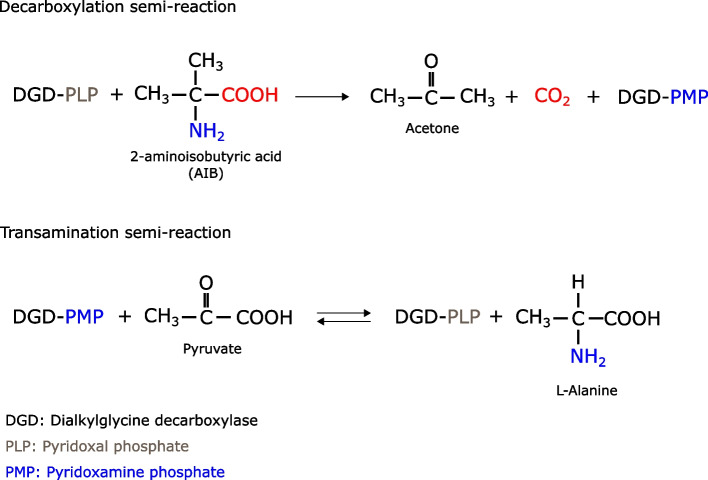
Dialkylglycine decarboxylase reaction. The aminotransferase catalyzes the AIB through two half-reactions. The first step corresponds to AIB decarboxylation releasing CO_2_. The second, to the transamination of the amino group of the intermediate PMP to the pyruvate that produces L-alanine [13, 14]

Dialkylglycine decarboxylase (DGDA) has first been described in the bacterium *Burkholderia cepacia* [8], and later in some fungi [9, 10]. This enzyme belongs to the aminotransferase class III, which uses either α-ketoglutarate or pyruvate as the preferred amino group acceptors, and the pyridoxal phosphate (PLP) as a cofactor, that covalently binds to the amino group of their substrates, which is normally located at end of the alkyl chain and generally distal to the carboxylate group. However, DGDA is one of the few exceptions to this aminotransferase family that binds to the alpha-amino group [11]. The enzyme exhibits the unique ability to catabolize the non-proteogenic dialkylamino acid 2-aminoisobutyric acid (AIB), which is present in soils and is an important component of the fungal antimicrobial oligopeptides called peptaibols [8, 12]. DGDA catalyzes AIB through two half-reactions (Fig. 1): (1) The AIB binds to the PLP pyridoxal ring to form an external aldimine followed by a decarboxylation reaction (first half-reaction) which releases CO_2_. (2) Then the formed quinonoid intermediate is reduced to form pyridoxamine phosphate (PMP) and an acetone molecule is released. (3) The PMP suffers a transamination reaction (second half-reaction) in which its amino group is transferred to a pyruvate molecule to form alanine and recycle the PLP [13].

In addition, we also identified a putative regulator of *DGD1*, named *DGD2*, that contains the characteristic functional motif of the zinc binuclear proteins. These proteins are known to be exclusive to fungi and are involved in the regulation of various physiological processes such as the induction of genes necessary for the metabolism of specific amino acids, for example, the well-reported regulator proteins Cha4p, Aro80p, and Leu3p, among others [15, 16].

Phylogenetic analysis showed that genes *DGD1* and *DGD2* might have been acquired by HGT from a member of the *Zygosaccharomyces* genus. The findings and experimental validation of this study show for the first time the evidence of an HGT event in non-*S. cerevisiae* species are involved in their adaptation to natural environments, in which dialkyl amino acid-containing peptaibols are released by fungi during microbial warfare.

## Results

### Identification of a yeast dialkylglycine decarboxylase-like gene

**Fig. 2:**
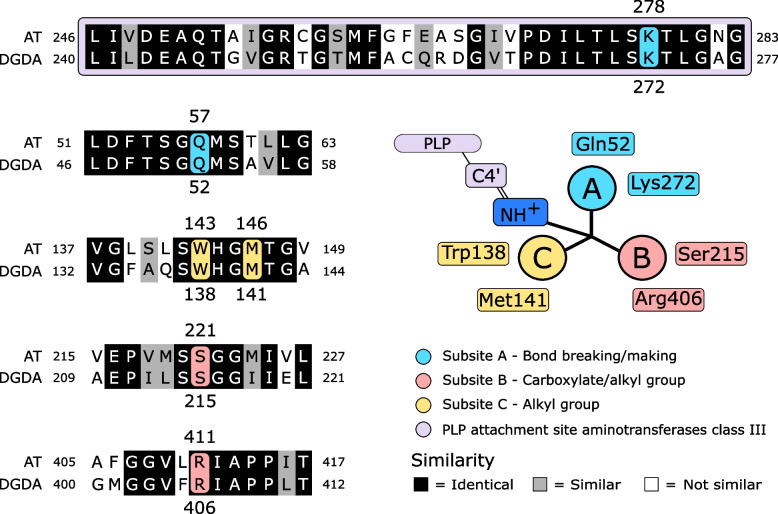
Dialkylglycine decarboxylase active site. The alignment between *Saccharomyces* AT and *B. cepacia* DGDA sequences shows that the PLP-binding domain (obtained from the PROSITE database) and residues involved in the catabolism of AIB are conserved in the deduced AT amino acid sequence. The scheme was adapted from [13, 14]

Reannotations of the genomes of *S. uvarum* CBS7001 and *S. kudriavzevii* IFO1802 revealed a novel cluster composed of three genes with unknown functions. This cluster is conserved between both *S. uvarum* and *S. kudriavzevii* genomes and is absent in the *S. cerevisiae* reference strain S288C. The blastx search of these three genes against the non-redundant protein sequence database revealed the presence of this cluster in other strains of *S. uvarum* and *S. kudriavzevii*, and in an additional *Saccharomyces* species, *S. eubayanus.* The absence of this cluster in the rest of the S*accharomyces* species was confirmed using the blastn algorithm against both the non-redundant database and *Saccharomyces* genome assemblies (Additional file 1: Table S1). The blastn search showed that the three genes may encode a putative zinc finger transcription factor (TF) similar to that encoded by the gene *CHA4*, an Aminotransferase-like protein (AT), and a putative amino acid permease (AAP). We obtain the deduced amino acid sequences from the AT-encoding genes of the *Saccharomyces* strains. The consensus sequence was used to define possible protein domains according to the PROSITE database. The analysis showed that AT contains a pyridoxal phosphate (PLP)-attachment site (Fig. 2) which belongs to the aminotransferase class III family [11]. The Blastp search of the predicted amino acid consensus sequence against the Swissprot database and a subsequent CLUSTAL Omega alignment showed that the AT shares 51.9% of identity with the *Burkholderia cepacia* dialkylglicine decarboxylase (DGDA) enzyme, whose substrate is the non-proteinogenic amino acid 2-aminoisobutyric acid (AIB) [8]. Moreover, the reported residues (Q52, W138, M141, S215, K272, and R406) to be involved at the subsites (A, B, and C) of the *B. cepacia* DGDA active site [14] are conserved in the yeast AT protein sequence (Fig. 2). Thus, this data suggests that the gene AT, hereafter referred to as *DGD1*, may encode a dialkylglicine decarboxylase enzyme, and those yeast strains carrying the gene *DGD1* could display a dialkylglycine carboxylase activity.

### Saccharomyces isolates with DGD1 can use 2-aminoisobutyric acid as a nitrogen source

**Fig. 3:**
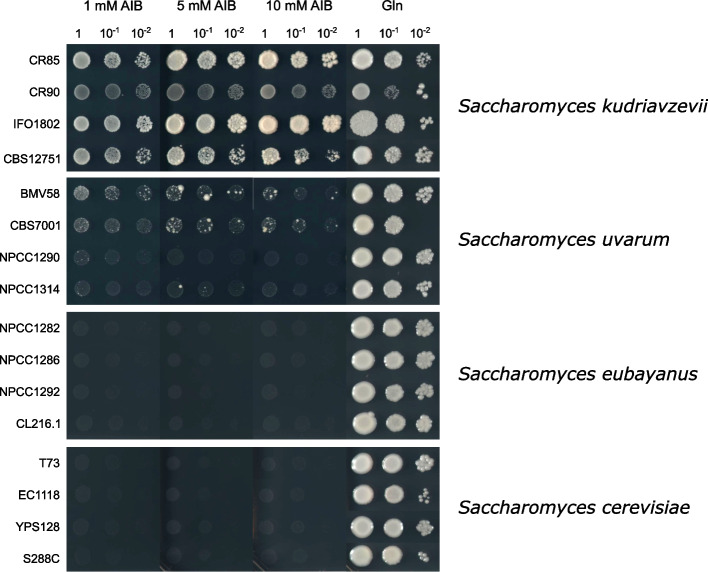
Growth screening of *Saccharomyces* isolates. The strains were cultured on YNB plates at the different AIB concentrations and 10 mM Glutamine (Gln) as the sole nitrogen source

Since it has been reported that *E. coli* expressing the *B. cepacia* DGDA gene acquired the ability to metabolize AIB [8] and *S. cerevisiae* is unable to use AIB nitrogen sources [17], we tested the ability of the *Saccharomyces* isolates carrying the gene *DGD1* (*S. eubayanus*, *S. uvarum*, and *S. kudriavzevii*) to use AIB as the sole nitrogen source. Therefore, we took different strains belonging to the species *S. eubayanus*, *S. uvarum*, *S. kudriavzevii*, and *S. cerevisiae* (Table 1) and plated them on YNB solid media at different AIB concentrations (1 mM, 5 mM, and 10 mM). After 11 days, the *S. kudriavzevii* strains IFO1802 and CR85 and, to a lesser extent, CR90 and CBS12751, were able to grow in the presence of AIB, and at major concentrations, a better growth was observed especially in the strain IFO1802 (Fig. 3, third column). The *S. uvarum* strains BMV58 and CBS7001 grew very poorly, but better than NPCC1290 and NPCC1314 (Fig. 3). However, none of the *S. eubayanus* strains were able to grow in these conditions. As expected, no growth was observed in the *S. cerevisiae* strains and all the *Saccharomyces* isolates exhibited optimal growth in the presence of glutamine after 4 days (Fig. 3, fourth column). These results demonstrated that some *Saccharomyces* isolates carrying the gene *DGD1* can grow in the presence of AIB, with variable growth rates depending on the species and strain. These results suggested that the novel *DGD1* gene may be responsible for the use of AIB as a nitrogen source.

**Table 1:** Wild and reference strains used in this study

Strain	Species	Source	Reference
T73	*S. cerevisiae*	Wine (Alicante, Spain)	[18]
EC1118	*S. cerevisiae*	Wine (Madrid, Spain)	Lallemand
YPS128	*S. cerevisiae*	*Quercus alba* (Pennsylvania, USA)	[19]
S288C	*S. cerevisiae*	Lab strain	[20]
BMV58	*S. uvarum*	Wine (Spain)	[21]
CBS7001	*S. uvarum*	*Mesophylax adoperus* (Spain)	[22]
NPCC1290	*S. uvarum*	*Araucaria araucana* (Tromen, Argentina)	[23]
NPCC1314	*S. uvarum*	Chicha (Pucón, Chile)	[24]
CR85	*S. kudriavzevii*	*Quercus ilex* (Ciudad Real, Spain)	[25]
IFO1802	*S. kudriavzevii*	Partially decay leaf (Japan)	[26]
CR90	*S. kudriavzevii*	*Quercus faginea* (Ciudad Real, Spain)	[25]
CBS12751	*S. kudriavzevii*	Soil (Taoyuan & Kaohsiung, Taiwan)	[27]
NPCC1282	*S. eubayanus*	*Araucaria araucana* (Cavihaue, Argentina)	[23]
NPCC1286	*S. eubayanus*	*Araucaria araucana* (Cavihaue, Argentina)	[23]
NPCC1292	*S. eubayanus*	*Araucaria araucana* (Tromen, Argentina)	[23]
CL216.1	*S. eubayanus*	*Nothofagus pumilio* (Villarrica, Chile)	[28]
CLIB830^T^	*T. microellipsoides*	Apple Juice (Bischofszell, Germany)	[29]
CBS6141	*T. microellipsoides*	Exudate of sandalwood (Hawaii, USA)	[29]
CBS6762	*T. microellipsoides*	Lemonade (Switzerland)	[29]
CBS6143	*T. microellipsoides*	Tea-beer (Finland)	[29]
NRRLY 17,058	*T. microellipsoides*	Tea-beer (Finland)	[29]
YB4811	*Z. kombuchaensis*	Kombucha tea (Rusia)	[30]
CECT1232	*Z. rouxii*	Black grape must (Italy)	[31]
CECT1924	*Z. baili*	Unknown (Japan)	[32]

### DGD1 confers the ability to use AIB as a nitrogen source

**Fig. 4:**
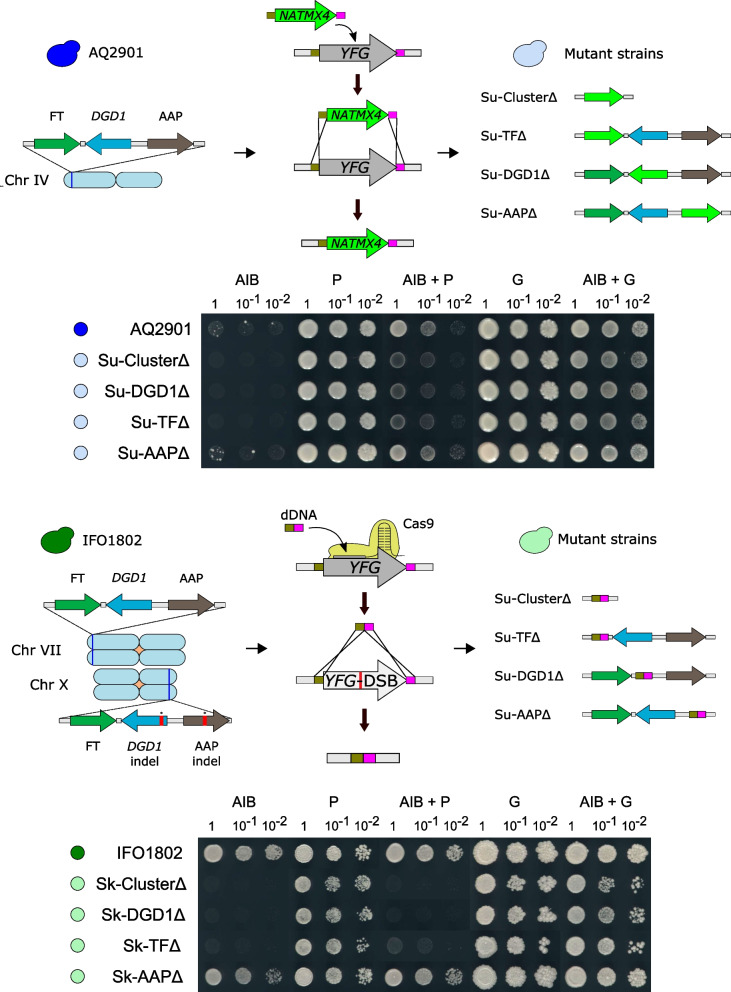
Growth and resistance against AIB phenotypes of the mutant strains. Above, the chromosome localization of the subtelomeric genes and the applied gene disruption approach to both strains IFO1802 and AQ2901 are shown. Below, the ability to use AIB as the sole nitrogen source (column AIB) and the resistance against the AIB’s inhibitory growth effect (columns P, AIB + P, G & AIB + G) exhibited by parental and mutant strains are depicted. G: 10 mM glutamine; P: 10 mM proline; AIB: 10 mM 2-aminoisobutyric acid

To test whether the gene *DGD1* is responsible for the dialkylglycine decarboxylase activity or not, we generated mutant strains of the entire cluster and mutants of each gene in both BMV58 and IFO1802 backgrounds, which respectively showed the best phenotypes among the tested *S. uvarum* and *S. kudriavzevii* isolates (Fig. 3). Moreover, the *S. kudriavzevii* IFO1802 strain contains two copies of these subtelomeric genes, located at chromosomes VII and X. The latter carries *DGD1* and APP alleles containing one indel that completely changes the reading frame and produces premature stop codons (Fig. 4). Once we generated the mutant strains (Table 2), they were cultured on YNB 10 mM AIB plates. Due to the slow growth rate exhibited by the *S. uvarum* strain BMV58 in the tested conditions, we also assayed the resistance against the growth inhibitory effect exerted by AIB [17]. We cultured the cells in the presence of AIB with either 10 mM glutamine or 10 mM proline. Additionally, we used either 10 mM glutamine or 10 mM proline as the sole nitrogen source as a control medium (Fig. 4). Glutamine, as a preferred nitrogen source, activates the nitrogen catabolite repression (NCR), a wide transcriptional regulation program enabling *Saccharomyces* yeast to repress genes involved in the utilization of poor and rare nitrogen sources when preferred ones are available. However, proline, as a poor nitrogen source, does not activate NCR. Among the repressed genes by NCR is *GAP1*, encoding a general amino acid permease involved in the uptake of all L-amino acids, some D-amino acids, related compounds, toxic analogs, and polyamines, likely including AIB.

**Table 2:** Engineered strains used in this study

Strain	Genotype	Reference
AQ2901	*MAT*a *ho::MX4dsdA*	This study
Su-ClusterΔ	*MAT*a *ho::MX4dsdA cluster*Δ*::NATMX4*	This study
Su-DGD2Δ	*MAT*a *ho::MX4dsdA dgd2*Δ*::NATMX4*	This study
Su-DGD1Δ	*MAT*a *ho::MX4dsdA dgd1*Δ*::NATMX4*	This study
Su-GAP1dΔ	*MAT*a *ho::MX4dsdA gap1d*Δ*::NATMX4*	This study
Su-ALT1Δ	*MAT*a *ho::MX4dsdA* alt1Δ*::KANMX*	This study
Sk-ClusterΔ	*cluster*Δ*::*Cluster-dDNA	This study
Sk-DGD2Δ	*dgd2*Δ*::*DGD2-dDNA	This study
Sk-DGD1Δ	*dgd1*Δ*::*DGD1-dDNA	This study
Sk-GAP1dΔ	*gap1d*Δ*::*GAP1d-dDNA	This study
Sk-ALT1Δ	*alt1*Δ::ALT1-dDNA	This study
CML235	*MAT*a *ura3-52 leu2*Δ*1 his3*Δ*200 TRP1 GAL2*	[33]
CML235-DGD1-Sk	*MAT*a *ura3-52 leu2*Δ*1 his3*Δ*200 TRP1 GAL2*; pYES-Sk-*DGD1*	This study
CML235-DGD1-Su	*MAT*a *ura3-52 leu2*Δ*1 his3*Δ*200 TRP1 GAL2*; pYES-Su-*DGD1*	This study
CML235-LacZ	*MAT*a *ura3-52 leu2*Δ*1 his3*Δ*200 TRP1 GAL2*; pYES-*LacZ*	This study

As we demonstrated above, the haploid *S. uvarum* BMV58-derived strain AQ2901 grew also after 11 days in the presence of AIB as the sole nitrogen source (Fig. 4, AIB column). However, the deletant strains Su-ClusterΔ and Su-DGD1Δ were unable to grow in this condition (Fig. 4, AIB column). Additionally, these strains showed decreased resistance against AIB after 4 days (Fig. 4, AIB + P column). But in the presence of glutamine, the inhibitory effect was negligible (Fig. 4, AIB + G column). As we expected, all the strains showed optimal growth on glutamine and proline as sole nitrogen sources (Fig. 4, P & G columns). We observed similar effects in the IFO1802 deletants Sk-ClusterΔ and Sk-DGD1Δ, which did not grow in the presence of AIB after 4 days (Fig. 4, AIB column). Additionally, the resistance against AIB was lost and hence no growth was observed in both mutants (Fig. 4, column AIB + P). Both Su-AAP∆ and Sk-AAP∆ mutants showed the same growth phenotype as their parental strains (Fig. 4). Surprisingly, the strains Su-TF and Sk-TF, which are mutants in the TF gene that encodes a putative Zing-finger transcription factor, showed similar phenotypes as the mutants Su-DGD1 and Sk-DGD1. Interestingly, the strain Sk-TF showed a little residual growth (Fig. 4, column AIB + P). These results suggest that gene *DGD1* might encode a dialkylglicine decarboxylase enzyme and the gene TF, from now named *DGD2*, could be involved in the regulation of the dialkylglycine activity.

### DGD1 encodes a yeast dialkylglycine decarboxylase enzyme

**Fig. 5:**
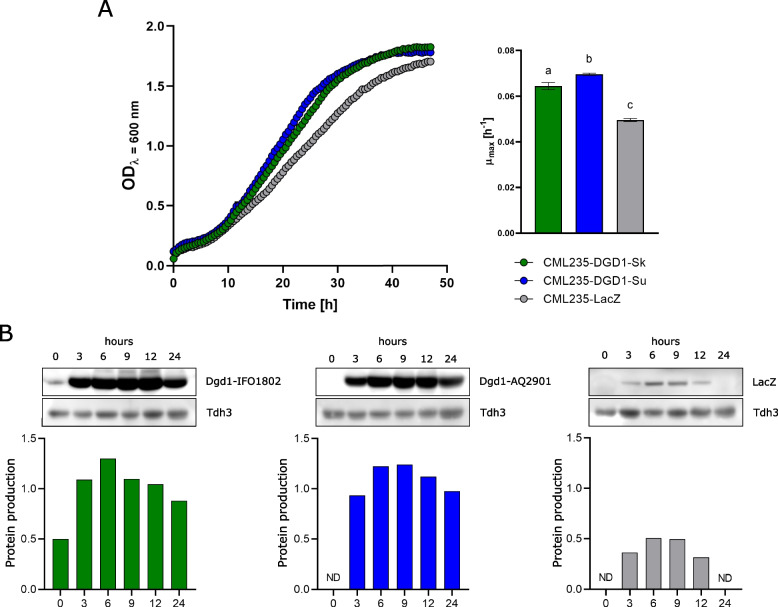
*DGD1*-carrying CML235 growth curves and Dgd1 protein production. **A** The cells were cultured in 200 µL of minimal medium (YNB, 10 mM AIB, 10 mM proline, 1% raffinose as carbon source, and 2% galactose as inducer) at 25 °C. On the right, a plot of the maximum specific growth rates (µmax) is shown. The error bars are standard deviations based on three biological replicas. Statistical differences were obtained through an ANOVA analysis. The raw data, Gompertz model fitting, and statistical analyses are given in Additional file 11. **B** Protein production profile at different time points after overnight cultures were transferred to the inducer medium. The V5-labelled Dgd1p and lacZ proteins were detected by horseradish peroxidase (HRP)-conjugated anti-V5 primary antibody. The loading protein control Tdh3p was detected by Anti-Tdh3p primary antibody coupled with an HRP-conjugated anti-mouse secondary antibody. Protein production is equal to the Dgd1p/LacZ peak band area divided by the Tdh3p peak band area. The Western blot raw data, the experimental molecular weight of the Dgd1p produced by the expression of the *S. uvarum* and *S. kudriavzevii DGD1* alleles, and the molecular weight calibration are given in Additional file 11

**Fig. 6:**
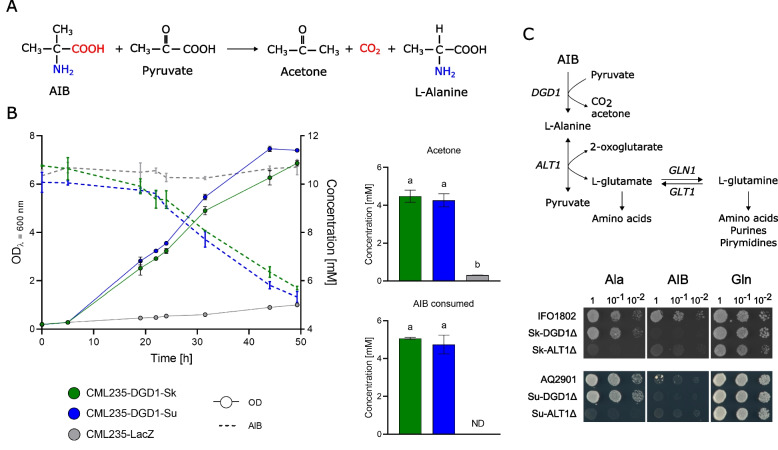
*Saccharomyces *in vivo Dgd1 activity. **A** Decarboxylation half-reaction is carried out by dialkylglycine decarboxylase [13, 14]. **B** Growth and AIB consumption curves showed by the induced protein producer strains (left), AIB consumption, and acetone production after 48 h (right). **C** *alt1* IFO1802 mutant is unable to grow in the presence of AIB. Ala: 10 mM L-alanine; Gln: 10 mM L-glutamine; AIB: 10 mM 2-aminoisobutyric acid. The error bars are the standard deviation of three biological replicas. Statistical differences were obtained through one-way ANOVA analysis. The raw data and statistical analyses are given in Additional file 12

To confirm the gene *DGD1* encodes a dialkylglycine decarboxylase enzyme, we cloned both *S. kudriavzevii* IFO1802 and *S. uvarum* AQ2901 *DGD1* genes (Additional files 2, 3, 4 and 5: Figs. S1-S4, respectively) into the expression plasmid pYES2.1 TOPO® TA to construct the plasmids pYES-Sk-DGD1 and pYES-Su-DGD1 (Table 4). They were used to transform the host *S. cerevisiae* lab yeast CML235 to generate the strains CML235-DGD1-Sk and CML235-DGD1-Su, respectively (Table 2). When both yeasts were cultured in a minimal medium with AIB (Fig. 5A), they showed an increment of about 30–40% of the µmax compared to the control strain CML235-LacZ. We also assayed the protein production kinetics by Western blot and observed a high protein production after 3 h of growth in the inducer medium, which was maintained constant until 24 h in the strains CML235-DGD1-Sk and CML235-DGD1-Su and 12 h for CML235-LacZ (Fig. 5B). Then, we quantified the transformation of AIB to acetone, the by-product of the decarboxylation half-reaction (Fig. 6A). We cultured the strains in the presence of AIB and we demonstrated that both CML235-DGD1-Sk and CML235-DGD1-Su strains consume AIB, and after 48 h they produced stoichiometric amounts of acetone compared to the *S. cerevisiae* lab strain CML235-LacZ (Fig. 6B). Moreover, to demonstrate that the end product of the Dgd1p reaction is alanine, and then incorporated into the nitrogen metabolism, we disrupted the alanine aminotransferase gene *ALT1* [34] in both *S. kudriavzevii* IFO1802 and *S. uvarum* AQ2901 strains, since it showed the best growth phenotype among all tested *Saccharomyces* isolates. Indeed, the mutant Sk-ALT1Δ showed a huge growth reduction in the presence of AIB. However, normal growth was observed in the mutant Sk-DGD1Δ growing in the presence of alanine (Fig. 6C). This confirms that the consumption of AIB as a nitrogen source follows the proposed pathway (Fig. 6C). All these results confirm that the gene *DGD1* encodes a yeast dialkylglycine decarboxylase.

### DGD2 encodes a putative zinc-cluster regulatory protein

Since the strains Sk-TF and Su-TF (hereafter named Sk-DGD2 and Su-DGD2) lost their ability to grow in the presence of AIB and showed an impaired resistance against AIB (Fig. 4), we hence analyzed the sequence of the novel gene *DGD2*.

**Fig. 7:**
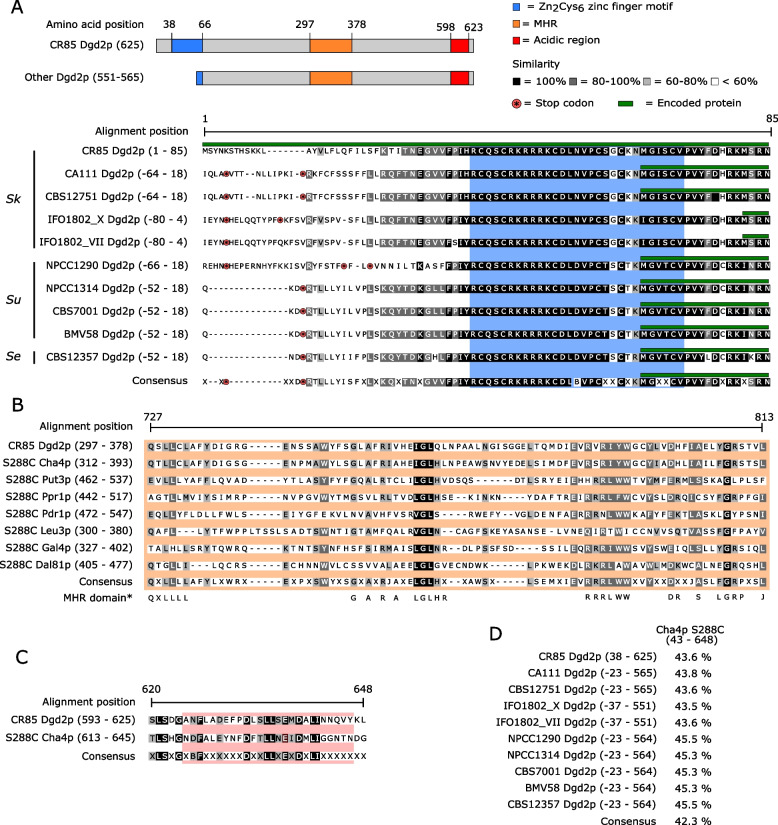
Dgd2p functional region determination. **A** Up, a general view of the positions of the identified functional sites in the CR85 Dgd2p compared to the other Dgd2p. Down, alignment of the available *Saccharomyces* Dgd2p amino acid sequences deduced from the orthologous nucleotide sequence, using CR85 as a reference. The upper green lines of each sequence indicate the encoded Dgd2p sequences according to the gene annotations. It can be observed that in most strains the methionines of the start codons are located 60–74 codons downstream of the start codon of CR85. When this homologous region of 60–74 codons is translated in the other strains, the start codon of CR85 is absent, and stop codons appear, indicating that this region is pseudogenized in all strains except CR85. **B** Alignment of the middle homology region (MHR) of *S. cerevisiae* zinc binuclear proteins with the CR85 Dgd2p sequence. (*) The conserved MHR amino acids were described elsewhere [35]. **C** Alignment of the *S. cerevisiae* Cha4p activation domain (AD) with the CR85 Dgd1p C-terminal region. **D** Identity of each *Saccharomyces* Dgd2p and the consensus amino acid sequence with respect to *S. cerevisiae* Cha4p. The numbers in the brackets indicate the amino acid positions of Chap4p referred to the start codon of CR85 Dgd2p

We first observed that the lengths of the different deduced amino acid sequences derived from the alleles were variable, between 551 and 625 residues (Fig. 7A). CR85 possesses the larger Dgd2p sequence and is the only one exhibiting a distinguishable fungal zinc finger Zn_2_Cys_6_ motif at the Nt end, according to the PROSITE database. Therefore, we performed a manual search upstream of the annotated start codons of the other available genome sequences of the *S. kudriavzevii* (CBS12357, CA111, CBS12751, and IFO1802) and *S. uvarum* strains (NPCC1290, BMV58, CBS7001, and NPCC1314) with shorter coding regions, and, despite these regions contained a putative Zn_2_Cys_6_ motif similar to that of CR85, the absence of start codons before the domain and the presence of stop codons in this region for all sequences indicated that these regions are pseudogenized in all strains except CR85 (Fig. 7A). To confirm this observation, we sequenced the DNA region corresponding to the zinc finger Zn_2_Cys_6_ of the *S. kudriavzevii* strains IFO1802 and AQ2901 and confirmed the presence of stop codons in that region (data not shown). Therefore, due to the pseudogenization of the 5′ end of the *DGD2* gene of all strains, except CR85, their Dgd2p proteins are translated from other downstream methionine start codon, resulting in shorter Dgd2 proteins lacking the Zn_2_Cys_6_ motif at the amino end.

In addition, the alignment of the CR85 Dgd2p with other *S. cerevisiae* zinc binuclear cluster proteins (the closely related Cha4p, as well as Put3p, Prpr1p, Pdr1p, Leu3p, Gal4p, and Dal81p) allowed us to identify the core part of the middle homology region motif (MHR) [35] located at the position 297–378 of Dgd2p (Fig. 7B). Moreover, Dgd2p also contains a region at C-terminal similar to the Cha4p activation domain (AD) (Fig. 7C) [35, 36]. Besides, the consensus Dgd2p, starting from the zinc finger motif sequence, showed 42.3% identity with the *S. cerevisiae* Cha4p. Identity was even higher in the individual Dgd2p sequences (Fig. 7D). Although the Zn_2_Cys_6_ is absent in most strains, the MHR and AD domains are present in Dgd2p from all *S. uvarum* and *S. kudriavzevii* strains. Thus, the above data suggest that the gene *DGD2* might encode a putative regulatory protein that specifically regulates the expression of *DGD1* in the presence of AIB, but regulation may be different in CR85 for the rest of the strains.

### The gene DGD2 is required to induce the expression of the DGD1 gene in the presence of 2-aminoisobutyric acid

**Fig. 8:**
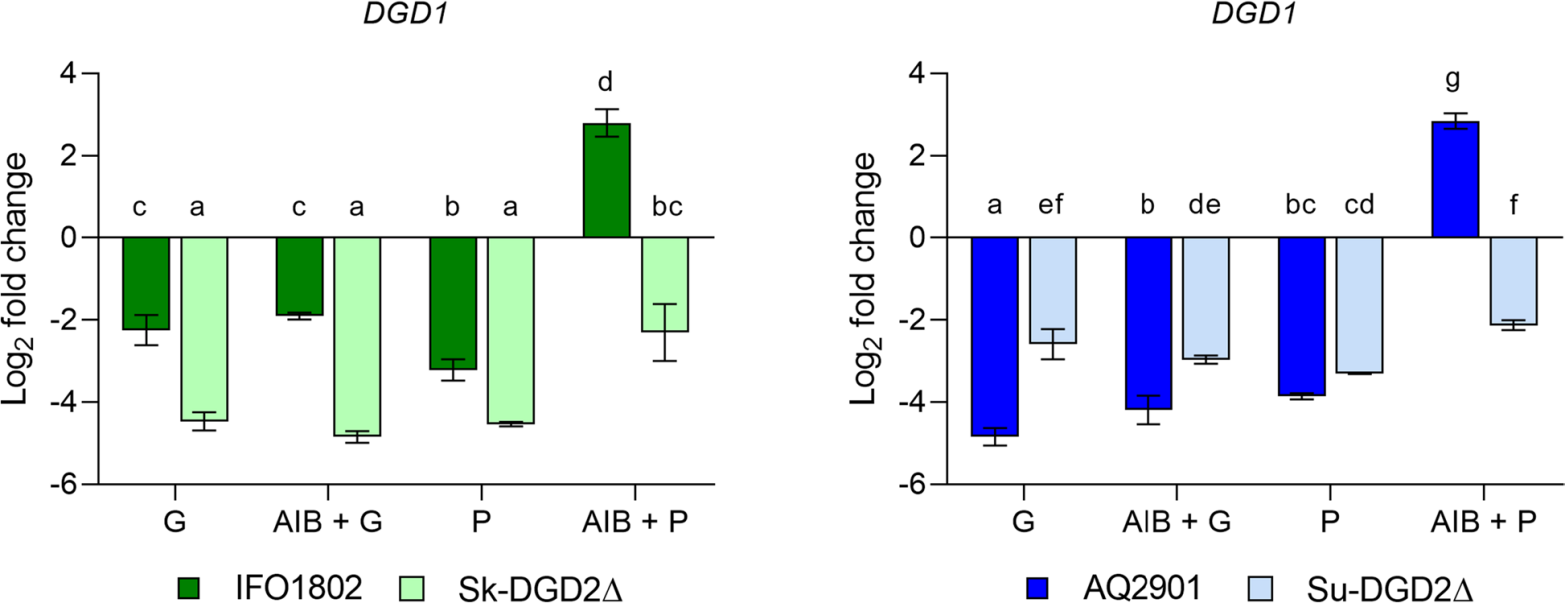
*DGD1* relative expression. The expression values of *DGD1* in the strains IFO1802 and AQ2901 and their *dgd2* mutants Sk-DGD2∆ and Su-DGD2∆ for each treatment are shown. The fold change represents the log-2 base of the relative expression of *DGD1*, normalized by the average expression of the reference genes *ACT1* and 18S rRNA divided by the average of the relative expression of all treatments. G: 10 mM Glutamine; P: 10 mM Proline; AIB: 1 mM 2-aminoisobutyric acid. The error bars are the standard deviation of three biological replicas. Statistical differences were obtained through 2-way ANOVA analysis. The raw and relative expression data, and statistical analyses are given in Additional file 13

To validate the function of Dgd2p as the regulator of *DGD1*, we cultured the wild-type strains *S. kudriavzevii* IFO1802 and *S. uvarum* AQ2901, and their *dgd2* mutants Sk-DGD2Δ and Su-DGD2Δ in a minimal medium with AIB. We previously determined that 1 mM AIB was the optimal concentration to culture both Sk-DGD2Δ and Su-DGD2Δ strains to reduce the impact of its growth inhibitory effect (Additional file 6: Fig. S5). When cell concentrations reached 1 × 10^7^ cells/mL, we extracted the total RNA to generate cDNA, to quantify the relative expression of *DGD1* by qPCR, using genes the actin gene *ACT1* and 18S rRNA as references. We observed a 65-fold and 104-fold induction of *DGD1* in the *S. kudriavzevii* IFO1802 and *S. uvarum* AQ2901 strains when they were cultured in the AIB medium plus proline compared to the value expression obtained in proline (Fig. 8, AIB + P vs P). These results confirm a specific induction of *DGD1* in the presence of AIB. However, we observe slight and no differences between the expression values obtained in AIB plus glutamine and glutamine in the strains AQ2901 and IFO1802, respectively (Fig. 8, AIB + G vs G). Interestingly, we observed a 26-fold and 128-fold induction in the AIB plus proline condition compared with the AIB plus glutamine condition in the strains IFO1802 and AQ2901, respectively (Fig. 8, AIB + G vs AIB + P). The latter result agrees with an inducer exclusion of *DGD1*. Finally, the ability to induce the expression of *DGD1* in the presence of AIB is lost in both mutant strains Sk-DGD2Δ and Su-DGD2Δ, in comparison to their parental strains IFO1802 and AQ2901, which showed a 33-fold and 31-fold induction of *DGD1*, respectively (Fig. 8, AIB + P). These results confirm that the gene *DGD2* encodes the positive regulator of *DGD1* required to induce the AIB-dependent expression of *DGD1*.

### On the origin of the Saccharomyces genes DGD1 and DGD2

**Fig. 9:**
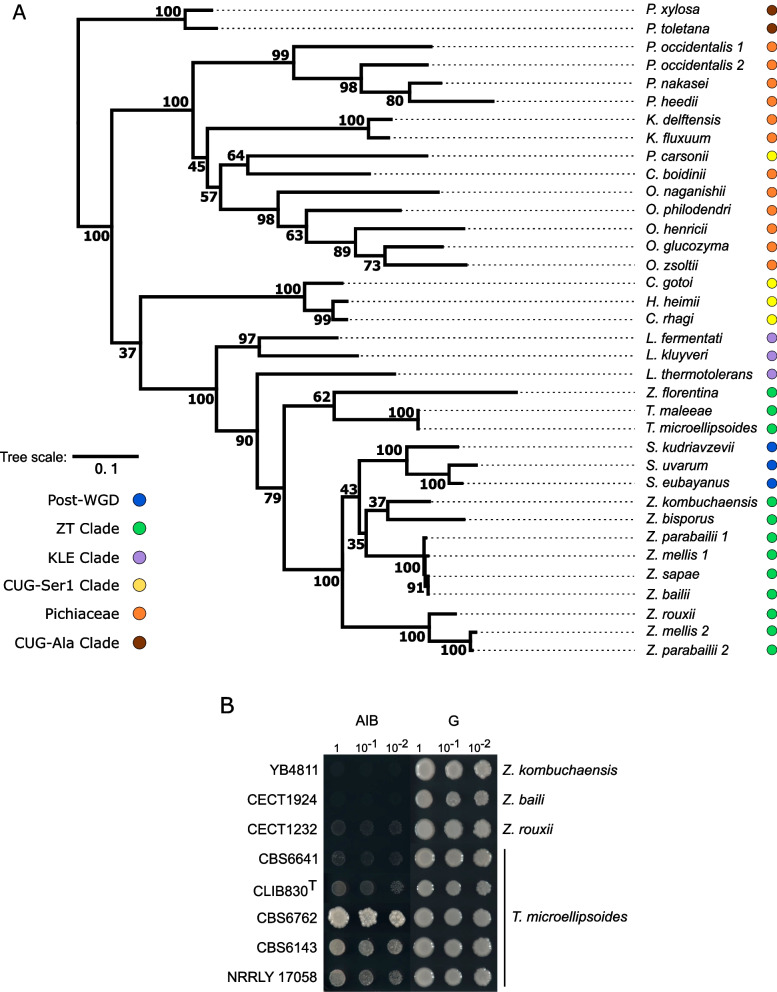
*DGD1* is present in other Saccharomycotina yeasts. **A** *DGD1* phylogenetic tree, inferred by using the maximum likelihood method conducted in MEGA 11, based on the LG model. The tree with the highest log likelihood (− 11,476.87) is shown. A discrete Gamma distribution was used to model evolutionary rate differences among sites (+ G, parameter = 0.5623). The tree is drawn to scale, with branch lengths measured in the number of amino acid substitutions per site. Numbers on the nodes correspond to bootstrap values based on 500 replicates. Circles of different colors at the right of the yeast names indicate the phylogenetic clades they belong to according to previous yeast phylogenetic analyses and taxonomic proposals [4, 37]. **B** Growth phenotype of *Zygosaccharomyces* and *Torulaspora* isolates in the presence of AIB as the sole nitrogen source

We identified the function of the genes *DGD1* and *DGD2*, which are needed to catabolize AIB. Since the dialkylglycine decarboxylase enzyme has been identified in bacteria and filamentous fungi [8–10], we searched for orthologues of the three genes *DGD1*, *DGD2*, and APP of the subtelomeric cluster in the available 313 genome sequences of different species of the subphylum Saccharomycotina [4] to subsequently perform phylogenetic and synteny analyses (Fig. 9A and Additional file 7: Fig. S6). In the case of the yet-unidentified AAP gene, we could not find orthologues nor possible related genes adjacent to *DGD1* or *DGD2*. In this way, we should remark that the presence of orthologous genes is conclusive, but their absence is not. Except in the case of highly accurate long-read sequencing of some of the *Saccharomyces* species, these genes can be unrepresented in the genome assemblages based on short-read sequencing due to their subtelomeric position or, in the case of genomes from yeasts belonging to lineages very distant to *Saccharomyces*, could be missed during the search due to low levels of similarity.

Except for the three *Saccharomyces* species under study, we found that *DGD1* (Fig. 9A) was only found in different yeast species belonging to lineages that diverged before the whole-genome duplication (pre-WGD) [38]. Moreover, *Saccharomyces DGD1* genes clustered together with members of the *Zygosaccharomyces/Torulaspora* (*ZT*) clade [39], instead of forming a differentiated clade (Fig. 9A), and maintaining the same *DGD1-DGD2* gene tandem order as *Z. kombuchaensis*, one of the closest species in the phylogeny (Additional file 7: Fig. S6), with an average divergence of 0.26 ± 0.01 nucleotide substitutions per site, compared to 0.30 ± 0.01 within *Zygosaccharomyces*, and 0.29 ± 0.01 between *Saccharomyces* and *Zygosaccharomyces*. The absence of this gene in post-WGD species, except for the three *Saccharomyces* species, the unexpected phylogenetic relationships within the ZT clade, and the conserved gene cluster synteny are evidence of a putative horizontal gene transfer event in the past.

In the case of the gene *DGD2*, the search for homologous genes in the available Saccharomycotina genome sequences resulted in the identification of *CHA4* and *DGD2* sequences due to their similarities, Cha4p and Dgd2p from Saccharomyces species showed a 44.8 ± 2.0% of average identity.

The subsequent phylogenetic analysis, based on their encoded proteins, showed that *CHA4* and *DGD2* are paralogous genes that were duplicated before the divergence of the Saccharomycetaceae yeasts (Additional file 7: Fig. S6). It is interesting to remark that the ancestral gene block order with *DGD1* has only been preserved in some *Lachancea* species, as *DGD1-CHA4*, and in *Z. kombuchaensis*, as *DGD1-DGD2*, even when the latter also contains a *CHA4* gene.

*CHA4* was preserved in most species of this family and the phylogenetic relationships shown by the *CHA4* gene clade are compatible with the *Saccharomycetaceae* species tree [4, 37, 40]. Contrastingly, the *DGD2* gene was only found in a few species of the ZT clade, *T. microellipsoides*, *Z. rouxi*, and *Z. kombuchaensis*. The Dgd2p of *Z. kombuchaensis* is the closest to the *Saccharomyces* Dgd2p (54.3% ± 1.9% of average identity), and this species is the only ZT clade taxon that maintains the same *DGD1-DGD2* synteny. These results reinforce the horizontal gene transfer hypothesis to explain the origin of the *Saccharomyces DGD1-DGD2* cluster.

Alternative explanations to the HGT (hypothesis 1): *DGD2* appeared as a duplication of *CHA4* in the ZT clade, and then transferred to *Saccharomyces* yeasts (hypothesis 2), or as a duplication of *CHA4* in the *Saccharomyces* genus and then transferred to the ZT clade species (hypothesis 3), were tested with the Shimodaira-Hasegawa [41], the one-sided Kishino-Hasegawa [42], and the Expected Likelihood Weight [43] methods based on likelihood estimates. Hypothesis 1 (lnL -37,378.68, *p*-values 1.000, 1.000, 0.957, respectively) was the best, and hypotheses 2 (lnL -37,411.72, *p*-values 0.036, 0.026, 0.020, respectively) and 3 (lnL -37,448.18, *p*-values 0.000, 0.000, 0.000, respectively) were worse and rejected.

The massive, independent *DGD2* gene losses required to explain the absence of *DGD2* in Saccharomycestaceae species are compatible with different pseudogenization events, likely due to loss of function, as observed among the *Saccharomyces* species under study.

Because *Saccharomyces DGD1* genes clustered with pre-WGD yeast of the ZT clade, we tested if our available *Zygosaccharomyces* and *Torulaspora* isolates (Table 1) were able to grow in the presence of AIB as the sole nitrogen source. Thus, the *Zygosaccharomyces rouxii* CECT1232 isolate showed slow growth in the presence of AIB. In contrast, the *Torulaspora* isolates grew well in the presence of AIB (Fig. 9B), being strain CBS6762 the one that exhibited the best growth phenotype.

All these results suggest that many pre-WGD yeast species exhibit dialkylglycine decarboxylase activity and the *Saccharomyces DGD1* might have been acquired from an ancient horizontal gene transfer event from a member of the ZT clade.

## Discussion

In this study, we identified a novel subtelomeric *Saccharomyces* gene cluster found in the species *S. uvarum*, *S. kudriavzevii*, and *S. eubayanus*, composed of three genes, of which we could characterize two of them, named *DGD1* and *DGD2*. We demonstrated that these two genes are involved in the catabolism of the non-proteinogenic amino acid 2-aminoisobutyric acid (AIB). *DGD1* encodes a yeast dialkylglycine decarboxylase enzyme initially identified by the presence of an aminotransferase class III PLP-binding site [11], the high identity showed with the *Burkholderia cepacia* dialkylglycine decarboxylase gene (DGDA) [8], and the ability of the *Saccharomyces* isolates possessing the *DGD1* gene to grow in the presence of AIB as a sole nitrogen source, although their phenotypes were variable both at the interspecific and intraspecific levels.

Deletants derived from strains *S. kudriavzevii* IFO1802 and *S. uvarum* BMV58, exhibiting the best AIB growth, were generated to validate *DGD1* as the gene responsible for the alkylglycine decarboxylase activity. Because the BMV58 strain initially exhibited a slow growth, we also tested an alternative way to assay the dialkylglycine decarboxylase activity which is based on the ability of yeasts to resist the inhibitory growth effect exerted by AIB, as reported by Kim and Roon [17]. The impaired growth and null resistance against AIB exhibited by the mutants Sk-DGD1∆ and Su-DGD1∆, compared with their parental strains, indicate that AIB exerts toxicity inside the cell and support that the gene *DGD1* encodes a dialkylglycine decarboxylase activity involved in the metabolization of the toxic AIB. Concordantly, the inhibitory effect of AIB was strong in the presence of proline, a “poor” nitrogen source, but insignificant in the presence of glutamine, a preferred nitrogen source. This experiment also demonstrates that the general amino acid permease Gap1p mediates the uptake of AIB into the cell. In the presence of a poor nitrogen source, such as proline, Gap1p is transporting AIB, but in the presence of glutamine, a preferred nitrogen source, *GAP1* is repressed and the uptake of AIB is not possible [17, 44]. Differences in the toxicity susceptibility or the AIB uptake may also explain the differences observed in the growth of the different *Saccharomyces* strains when AIB was the sole nitrogen source.

Finally, AIB consumption and the acetone production from AIB, which is the by-product of the first decarboxylation half-reaction, also demonstrated that *DGD1* encodes a yeast dialkylglycine decarboxylase enzyme. Both AIB usage and acetone production specifically underline dialkylglycine decarboxylase activity [9, 10, 45]. Moreover, the reduced growth of the mutants Sk-ALT1Δ and Su-ALT1Δ in the presence of AIB confirmed that the genes involved in the use of AIB as a nitrogen source are *DGD1* and *ALT1*.

Surprisingly, we observed similar impaired phenotypes in the *dgd2* mutants. Therefore, we analyzed the deduced amino acid sequence of the protein encoded by *DGD2*. We found a zinc finger Zn_2_Cys_6_ motif, which is only conserved in the *S. kudriavzevii* CR85 strain but is absent in the other strain sequences according to their shorter 5′ end of their coding regions. In the other strains, the homologous Zn_2_Cys_6_ motif region is pseudogenized (absence of the original star codon and the presence of stop codons) and Dgd2p is translated from an alternative star codon located 60 codons (74 in the case of IFO1802) downstream of the original one. In *Saccharomyces*, genes located in subtelomeric regions are recombinogenic and subjected to a higher mutation rate, and hence, can become totally or partially pseudogenized [46].

Although the DBD’s metal biding portion is essential for the DNA binding activity, it can be dispensable in some cases and still exerts its regulatory activity [15]. In particular, the expression of a truncated-zinc Zn_2_Cys_6_ finger motif *DAL81* allele in a *dal81 S. cerevisiae *strain recovers its ability to induce the urea amidolyase activity, encoded by the Dal81p-regulated *DUR1*,*2* gene [47]. The same was observed when the expression of a truncated *DAL81* allele lacking the six cysteine residues of the Zn_2_Cys_6_ finger motif could recover the ability of a *dal81* mutant to express the β-galactosidase reporter under the control of the GABA upstream activation sequence (UAS_GABA_), which is present in the promoters of both Dal81p- and Uga3p-regulated genes *UGA1* and *UGA4* [48]. Similarly, the mutation of the fourth cysteine residue of the Zn_2_Cys_6_ finger motif in the *Aspergillus nidulans tamA* gene, which is homologous to the *S. cerevisiae DAL81* gene, did not impact on protein’s function, confirming that this motif is not required for its regulatory function [49]. The same behavior was observed in the regulatory protein Gcr1p, where the expression of a truncated *GCR1* allele lacking its DBD could recover the wild-type phenotype of a *gcr1* mutant [50]. In addition, the alignment of the CR85 Dgd1p sequence with other reported zinc binuclear cluster proteins allowed us to identify the characteristic functional domains shared by the members of this family [15, 35]. The middle homology region (MHR) domain in Dgd1p, which is believed to regulate the transcriptional activity of these proteins [35] and the region similar to the reported Cha4p activation domain (AD) at the C-terminal position, needed to recruit the transcriptional machinery into the promoter of the regulated target gene [51].

Despite the absence of the Zn_2_Cys_6_ finger motif, we demonstrated an AIB-dependent induction of the *DGD1* gene. The null induction observed in the wild-type strains grown in the presence of AIB plus glutamine instead of proline was probably because AIB is not being transported into the cell since glutamine represses the activity of the permease Gap1p [44], and hence, the AIB uptake. A similar phenotype was observed in the double mutant *gap1 agp1*, which is defective in the uptake of tryptophan into the cell, and showed an expression reduction of 75% of the Aro80p-regulated *ARO9* gene in the presence of its inducer tryptophan [52]. Therefore, the results agree with an inducer exclusion of *DGD1*, meaning that AIB must be uptaken into the cell before initiating the expression of *DGD1*. Besides, the inability to express *DGD1* in both *dgd2* mutants Sk-DGD2Δ and Su-DGD2Δ in the presence of AIB confirmed that *DGD2* is required for the AIB-dependent induction of *DGD1*.

The absence of the Zn_2_Cys_6_ motif in the tested strains (*S. kudriavzevii* IFO1802 and *S. uvarum* AQ2901), however, has arisen the question of how the encoded Dgd2p from these alleles regulate the expression of *DGD1*. Moreover, the poor growth and basal *DGD1* expression exhibited by the *dgd2* mutant suggest the involvement of other unidentified regulatory elements. We observed that the similarity between Dgd2p and the regulatory Cha4p was 43–45%. Cha4p requires the coactivator complexes SAGA and Swi/Snf to regulate its target genes *CHA1* and *SER3* in response to serine and threonine [36, 53]. The long segments conserved across Dgd2p compared to those of Cha4p suggest that this protein might interact with some of these complexes in the presence of AIB to induce the expression of *DGD1*. Besides, both Gcr1p and Dal81p, where their DBD is dispensable, bind as heterodimers with the target proteins Rap1 and Dal82, respectively. The two latter proteins confer the required DBD for binding into the promoters of their target genes [50, 54]. In addition, some members of the zinc binuclear protein family act as nutrient sensors, interacting either in or directly with the target nutrient such as the well-reported Gal4p and Put3p, respectively [16]. Therefore, a similar mechanism might be working on Dgd2p, which senses the intracellular AIB, producing a conformational change that allows the recruitment of the transcriptional machinery, but binds into the *DGD1* promoter as a heterodimer with a yet-unidentified protein that confers the DNA binding activity.

Although we characterized the function of the genes *DGD1* and *DGD2*, their origins and physiological roles in nature were unclear. We found orthologues of both genes in the other Saccharomycotina yeasts, where the synteny was conserved in several pre- and post-WGD clade species. In addition, we demonstrated that some isolates from the ZT and KLE clades exhibited dialkylglycine decarboxylase activity. Both *Saccharomyces DGD1* and *DGD2* clustered within the ZT clade, instead of forming their differentiated clade in contrast to the species phylogeny [4, 37, 40]. The unexpected phylogenetic position, the identical gene order, and the absence of these genes in other post-WGD species are clear evidence of a horizontal gene transfer event [1, 2, 5] from a member close to *Zygosaccharomyces*. Gene acquisition from non-*Saccharomyces* donors has also been reported in wine *S. cerevisiae* strains [3, 6], including the gene *FSY1* [7], which was also lost in the post-WGD species [55] and, later, horizontally transferred to *S. cerevisiae* [6].

A reported great source of AIB is the fungal antimicrobial oligopeptides known as peptaibols, which contain high levels of AIB (~ 56%), giving them a hydrophobic helix conformation, and therefore, increased stability and resistance against proteases [12, 56]. A variety of peptaibols has been identified in *Trichoderma* species, and many studies have proposed their use as biocontrol agents against pathogenic fungal microorganisms [57, 58].

Moreover, isovaline, another dialkylamino acid found in the structure of the peptaibols [12], was reported as an inducer of the DGDA activity [8], and can also be consumed by those yeasts with dialkylglycine decarboxylase activity (Additional file 8: Fig. S7). Since peptaibol-producing fungi are found in many natural niches [58] and *Zygosaccharaomyces* isolates are widespread in the same natural environments (soil, decayed leaves, and tree bark) as the wild *Saccharomyces* non-*cerevisiae* species [31], together with the findings obtained in this study, this prompted us to propose that these wild *Saccharomyces* yeasts acquired both *DGD1* and *DGD2* from an ancient member close to the *Zygosaccharomyces* genus, as part of detoxification mechanism through the catabolism of AIB, a by-product of the peptaibol degradation. Later, these genes could be lost in the evolutionary more recent *Saccharomyces* species, perhaps due to loss of function during the colonization of new habitats, such as sugar-rich substrates and fermentation environments where peptaibols are absent.

## Conclusions

In this study, we identified and characterized the novel *Saccharomyces* genes *DGD1* and *DGD2* which encode a yeast dialkylglycine decarboxylase enzyme and its AIB-responsive positive regulator, respectively, that are involved in the catabolism of AIB. The Dgd2p exerts its regulatory function despite the absence of its DBD motif which suggests the involvement of other regulatory proteins. The phylogenetic analysis indicated that both genes might have been acquired through HGT from a yeast closely related to the *Zygosaccharomyces* clade. Our results suggest that these genes could confer a detoxification mechanism against the inhibitory effect of AIB, which can be released as a potential degradation product of peptaibols produced by fungi in natural environments. Future experiments will address cis-regulatory elements of *DGD1*, the Dgd2p functional domain, and how the proposed regulatory proteins interact with Dgd2p to promote de AIB-dependent induction of *DGD1*.

## Methods

### Yeast strains

All the wild strains used in this study are listed in Table 1. These strains were grown on YPD solid medium plates (1% yeast extract, 2% peptone, 2% glucose, 2% agar) and maintained at 4 °C for further experiments.

### Identification of novel genes in Saccharomyces species

Both *S. uvarum* CBS7001 and *S. kudriavzevii* IFO1802 genomes [59] were re-annotated following a combination of two approaches as described elsewhere [60], which revealed a novel cluster of three unknown genes. A blastx search [61] of the three genes against the non-redundant protein sequence database was performed in other *S. uvarum* and *S. kudriavzevii* strains and *S. eubayanus* CBS12357 [62]. Simultaneously, a blastn search against both the non-redundant database and *Saccharomyces* assemblies (Additional file 1: Table S1) was carried out to check either the presence or absence of these genes. The discovered aminotransferase-like protein (AT) and putative zinc finger transcription factor (TF) gene sequences in the *S. kudriavzevii* strains IFO1802, CA111, CBS12751, and CR85; *S. uvarum* strains NPCC1290, NPCC1314, CBS7001, BMV58, and *S. eubayanus* strain CBS12357 were translated to their deduced amino acid sequence with the Geneious Prime® 2020.2.4 software using the standard code. The sequences were aligned with the Clustal Omega 1.2.2 tool using the default parameters, and a consensus sequence was extracted. A search for documented protein domains was performed through the InterProScan [63] tool of Geneious Prime® 2020.2.4 software against the PROSITE database and a blastp search against the Swissprot database was carried out with default parameters using a max E-value equal to 0.1. Then, we extracted the sequence with the highest identity, and we carried out a new alignment with the Clustal Omega 1.2.2 tool.

### Zinc finger DNA region sequencing

A 600-bp PCR product that covers the *DGD2* zinc finger motif DNA sequence (approximately 300 bp upstream from the annotated sequence) was amplified from total DNA extracted from AQ2901 and IFO1802 strains using Phusion™ High-Fidelity Polymerase (Thermo Scientific) following the provider instructions employing sequencing primers (Table S6). They were loaded into 2% agarose gel, rescued, and purified through MinElute® PCR purification Kit (Qiagen). The fragments were sequenced through the University of Valencia Experimental Research Support Central Service’s Sanger sequencing (SCSIEUV, Spain) using the sequencing primers.

### Growth phenotype screening

The *Saccharomyces* strains (Table 1) were used to assay their ability to grow in the presence of 2-aminoisobutyric acid (AIB) as the sole nitrogen source. Precultures were incubated overnight in liquid YNB medium (0.17% yeast nitrogen base without amino acid and ammonium, 2% glucose, 5 g/L (NH_4_)_2_SO_4_) at 25 °C. Then, cells were grown on YNB plates (0.17% yeast nitrogen base without amino acid and ammonium, 2% glucose, 2% agar) containing three different AIB (Sigma Aldrich, Saint Louis, MO) concentrations (1, 5, and 10 mM) at 25 °C. Growth on YNB solid media containing 10 mM glutamine (Sigma Aldrich, Saint Louis, MO) was used as a control.

### Usage of isovaline as a nitrogen source

IFO1802 wild-type strain and their *dgd1* and *dgd2* mutants were plated on YNB solid media containing 10 mM L-isovaline (Thermo Fisher Scientific, Waltham, MA, USA) either as the sole nitrogen or plus 10 mM proline (Aditional file 8: Fig. S7). The plates containing 10 mM proline, AIB, and AIB plus proline were used as control media.

### Mutant strain construction

The mutant strains generated, and the primers used in this study are listed in Tables 2 and 3, respectively. The *S. uvarum* strain AQ2901 is a haploid derivate of the commercial wine strain BMV58 (Lallemand, Montreal, Canada). Strains AQ2901 and IFO1802 were transformed using the lithium acetate method changing the thermal shock to 37 and 34 °C, respectively [64]. The deletions of the subtelomeric individual genes and the entire cluster in the AQ2901 strain were carried through PCR-mediated gene disruption using the *NATMX4* cassette as a selection marker [65]. The *NATMX4* cassette was PCR amplified from the pAG25 plasmid [66] using NZY*Taq* II DNA Polymerase (NZYTech, Lisbon, Portugal) following the supplier’s instructions and employing deletion primers (Table 3), whereas the cassette *KANMX* from the plasmid pUG6 [67] was used for the deletion of the *ALT1* gene and was amplified with the corresponding deletion primers (Table 3) under the same conditions mentioned above. Total DNA was extracted from antibiotic-resistant transformants which grew on a selective YPD solid medium (1% yeast extract, 2% peptone, 2% glucose, 2% agar, 100 µg/mL nourseothricin or 200 µg/mL G418) at 25 °C after 4 days and gene deletions were confirmed by PCR analysis using the diagnostic primers (Table 3).

**Table 3:**
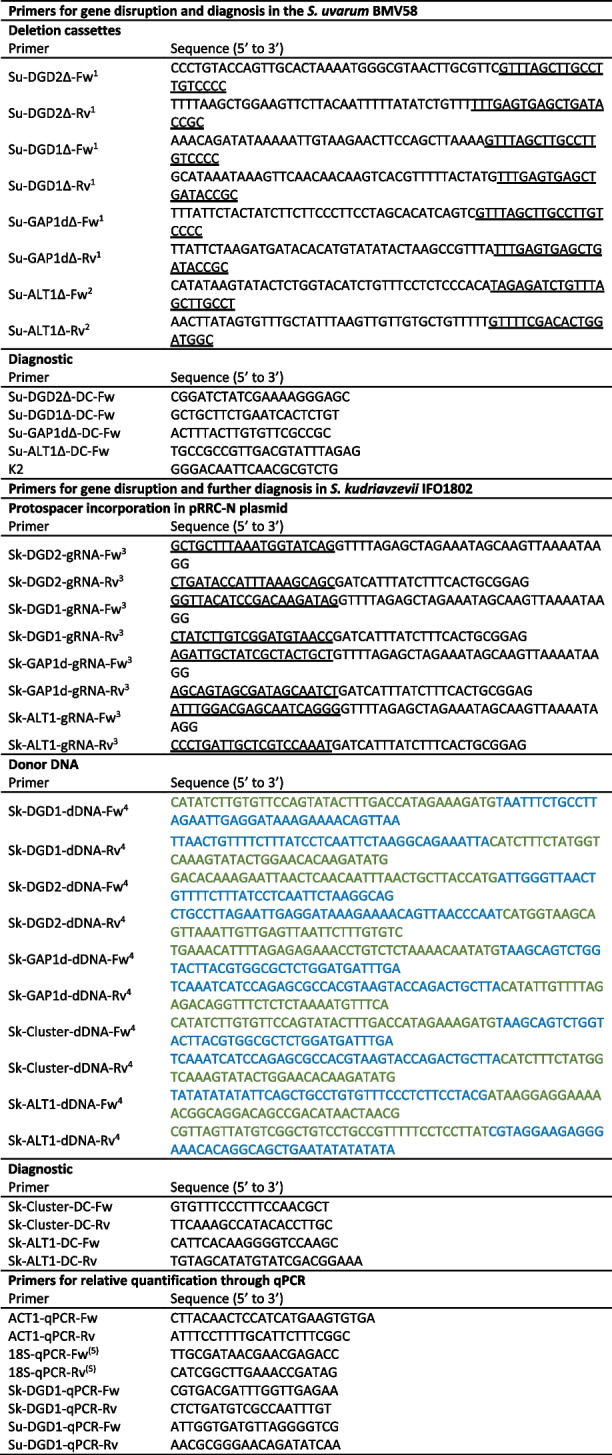
Primers used in this study

^1^Homologous sequences to *NATMX4* from the pAG25 plasmid are underlined.

^2^Homologous sequences to *KanMX* cassette from the pUG6 plasmid are underlined.

^3^The underlined sequences stand for the protospacers.

^4^Sequences homologous to both upstream and downstream sequences of the disrupted genes are labelled in green and blue, respectively.

^5^Primer sequences extracted from reference [21]

Deletion of the four copies of the subtelomeric genes and the two *ALT1* alleles in the *S. kudriavzevii* diploid strain IFO1802 was performed through CRISPR-Cas9 gene disruption [68]. The protospacer sequences were chosen according to [69], implementing the IFO1802 genome sequence as a reference to avoid selecting unspecific gRNA. Then, we amplified by PCR the whole pRCC-N plasmid by PCR which carries the *natMX* resistance marker with primers carrying the protospacer sequence at their 5′ ends [70]. The PCR was carried out with Phusion™ High-Fidelity Polymerase (Thermo Scientific, Vilnius, Lithuania) using the protospacer-carrying primers (Table 3). Before the addition to the transformation mix, 30 µL of the PCR product was treated with 10U of DpnI (Thermo Scientific, Vilnius, Lithuania) for 3 h to guarantee the total degradation of the pRCC-N original template. To ensure DNA reparation by homologous recombination, we used a double-stranded 80-bp oligonucleotide as donor DNA (dDNA), with 40 nucleotides on either side that are homologous to both upstream and downstream sequences of the target gene, respectively [71]. The oligonucleotides were assembled by mixing equal molar amounts of two complementary single-stranded 80-bp oligonucleotides (Table 3), heating the mix to 99 °C for 5 min, and subsequently, cooling down to 25 °C at a rate of 0.1 °C/s. One nanomole of the double-stranded oligonucleotide was added to the transformation mix, and the gene deletions were confirmed by PCR analysis using total DNA extracted from nourseothricin-resistant transformants, using the corresponding diagnostic primers (Table 3).

### Mutant strain phenotyping

The generated mutant strains of AQ2901 and IFO1802 (Table 2) were incubated overnight in a liquid YNB medium at 25 °C, and then, cultivated on YNB plates containing 10 mM AIB as the sole nitrogen source. The resistance against the AIB growth inhibitory effect was also assayed [17]. Briefly, cells were plated on YNB solid media containing 10 mM AIB plus either 10 mM proline (Sigma Aldrich Saint Louis, MO) or 10 mM glutamine. Growth on YNB plates containing only either 10 mM proline or 10 mM glutamine as the sole nitrogen source were used as controls. Similarly, the *alt1* mutant was incubated on YNB plates containing either 10 mM AIB, 10 mM L-glutamine, or 10 mM L-alanine (Sigma Aldrich Saint Louis, MO).

### DGD1 cloning

*DGD1* genes were amplified from the genomic DNA of IFO1802 and AQ2901 strains using the cloning primers (Additional file 9: Table S2). The PCR was carried out using TaKaRa Ex *Taq™* polymerase (Takara, Kusatsu, Shiga, Japan) following the supplier’s instructions. The *DGD1*-IFO1802 PCR product was purified through the MinElute® PCR purification Kit (Qiagen, Hilden, Germany). Before the cloning step, 1 µg of *DGD1*-IFO1802 PCR product was treated with 10 U *Xcm*I (New England BioLabs®, Ipswich, MA, USA) in NE Buffer 2.1 to digest the indel-containing *DGD1* copy amplified from chromosome X. The digestion product was loaded into an agarose gel, and the non-digested band corresponding to the chromosome VII copy was rescued and purified with the MinElute® PCR purification Kit (Additional file 2: Fig. S1). The DGD1-AQ2901 was purified with the High Pure PCR Purification Kit (Roche, Mannheim, Germany) to discard the small unspecific band (Additional file 3: Fig. S2). Both *DGD1* genes were inserted into the pYES2.1 TOPO® TA plasmid (Invitrogen, Waltham, MA, USA) and cloned into *E. coli* TOP10F’ strain (Invitrogen Waltham, MA, USA). The transformant *E. coli* strains (Additional file 9: Table S3) were selected on Ampicillin LB plates (1% tryptone, 0.5% yeast extract, 1% NaCl, 2% agar, 100 µg/mL ampicillin). The right orientation of the *DGD1* inserts was checked through the analysis of the restriction fragment length pattern obtained from the digestion of the *DGD1*-carrying plasmids (Table 4). The plasmids carrying the AQ2901 *DGD1* allele were digested with *Pvu*II (Fermentas Waltham, MA, USA) in buffer G, and those carrying the Chromosome VII IFO1802 allele were double digested with *Pvu*II and *Sph*I (Invitrogen, Waltham, MA, USA) in buffer G. Once the right *DGD1* orientation was confirmed in both plasmids (Additional files 4 and 5: Figs S3 and S4, respectively), the sequences of the inserts were obtained by Sanger sequencing using sequencing primers (Additional file 9: Table S2), at the Genomics Section of the Central Service of Experimental Research Support (SCSIE), University of Valencia, Spain. The haploid *S. cerevisiae* CML235 strain, which is a spore-derivative from the strain FY1679, was used as the host cell for recombinant protein production. The strain was transformed with 400 ng of the constructed plasmids pYES-Sk-*DGD1* and pYES-Su-*DGD1* (Table 4) using the lithium acetate method and the transformants were selected on a solid SC drop-ura medium (0.67% yeast nitrogen base without amino acid, 1.93% p/v synthetic complete drop-out [Formedium], 2% glucose, 2% agar) and the plasmid pYES-*LacZ* (Table 4) was used as the plasmid control.

**Table 4:** Plasmids used in this study

Plasmid	Comments	Reference
pAG25	*NATMX4*	[66]
pUG6	*KANMX*	[67]
pRRC-N	2µ origin, *natMX*, p*SNR52*-gRNA, p*ROX3*-*cas9*	[70]
pYES2.1 TOPO® TA	2µ origin, p*GAL1*-*CYC1* TT-V5 epitope-6xHis, *URA3*, *Amp*^r^, pUC ori, f1 ori	Invitrogen
pYES-LacZ	pYES2.1 TOPO® TA, *LacZ* ORF	Invitrogen
pYES-Sk-DGD1	pYES2.1 TOPO® TA, *DGD1*(IFO1802-ChrVII) ORF	This study
pYES-Su-DGD1	pYES2.1 TOPO® TA, *DGD1*(AQ2901) ORF	This study

### Growth phenotype of CML235 strains carrying the DGD1 genes

To test the growth phenotype in the presence of AIB of the *DGD1*-carrying CML235 strains (Table 2), overnight precultures of the strains CML235-DGD1-Sk, CML235-DGD1-Su, and CML235-LacZ were carried out in SC drop-ura medium containing 2% raffinose, as carbon source instead of glucose. Then, they were inoculated in 200 µL of YNB liquid medium containing 10 mM AIB, 10 mM proline, required amino acid supplements for auxotrophies (500 mg/L histidine, 125 mg/L leucine) [72], 1% raffinose as carbon source, and 2% galactose for inducing the expression of *DGD1*, with an initial OD_600_ of 0.2 on 96 well-microtiter plates at 25 °C. Growth curves were monitored through OD measurements at 600 nm wavelength in a SPECTROstar Nano® microplate reader (BMG LABTECH). Growth curves were fitted to the Gompertz model by using the grofit R package [73], and maximum specific growth rates (µmax) were obtained.

### Recombinant protein production kinetics

The strains CML235-DGD1-Sk and CML235-DGD1-Su were incubated in 15 mL of SC drop-ura containing 2% raffinose overnight at 30 °C, then the cells with an initial OD_600_ of 0.4 were inoculated in 100 mL of inducer medium (0.67% yeast nitrogen base without amino acid, 1.93% p/v synthetic complete drop-out, 1% raffinose, and 2% galactose) in 250-mL flasks at 30 °C with a shacking speed of 160 rpm. The strain CML235-LacZ was used as a recombinant protein control producer. The growth curves were followed at different times (0, 3, 6, 9, 12, and 24 h) through OD measurements at 600 nm wavelength using a Fisherbrand™ Cell Density Meter. Five-milliliter samples were taken, and the cell pellets were collected and frozen with liquid nitrogen and stored at − 80 °C until recombinant protein production analysis. Cell extracts were prepared according to a slightly modified post-alkaline extraction [74]. Briefly, cell pellets were resuspended in 200 µL of 0.2 M NaOH and incubated for 5 min at room temperature. Then they were pelleted and resuspended in 100 µL SDS-PAGE loading buffer (0.06 M Tris–HCL pH 6.8, 25% glycerol, 2% SDS, 14.4 mM β-mercaptoethanol, 0.1% bromophenol blue) and boiled for 5 min and pelleted again. Equal amounts of proteins were loaded and resolved in 12% SDS-PAGE gels and transferred to nitrocellulose membranes. Ponceau staining was used to confirm the correct protein transfer. The recombinant V5-tagged Dgd1 proteins were detected employing a horseradish peroxidase (HRP)-conjugated anti-V5 primary antibody (R96125; Invitrogen, Waltham, MA, USA). Anti-Tdh3p primary antibody (provided by Daniel Gonzalbo, University of Valencia) and HRP-conjugated anti-mouse secondary antibody (GE Healthcare Life Sciences, Chicago, IL, USA) were used to determine Tdh3p protein levels as the loading control. Pierce™ ECL Western Blotting Substrate (32,109, Thermo Scientific Waltham, MA, USA) was used for the detection of HRP-labelled proteins. Immunoblot images were obtained in an Amersham ImageQuant 800 system (Cytiva, Marlborough, MA, USA). Specific signals and peak band area quantifications were determined with ImageQuant TL software. Relative quantification of recombinant Dgd1p proteins at each time was calculated as the Dgd1p peak band area divided by the Tdh3p peak band area.

### In vivo Dgd1p activity assay

Strains CML235-DGD1-Sk, CML235-DGD1-Su, and CML235-LacZ were incubated overnight in 20 mL of SC drop-ura containing 2% raffinose at 30 °C. Afterward, cell pellets were centrifugated at 3500 × *g* and the supernatant was discarded. Then, pellets were resuspended in 20 mL of inducer medium for 3 h. At that moment, the cell pellets were recovered again and washed once in 20 mL of YNB-AIB medium (10 mM AIB, 500 mg/L histidine, 125 mg/L leucine, 1% raffinose, and 2% galactose) and inoculated in 100 mL of YNB-AIB medium with an initial OD_600_ of 0.2 at 30 °C with 120 rpm of shacking. Growth curves were followed at different times through OD measurement at 600 nm wavelength using a Fisherbrand™ Cell Density Meter (Fisher Scientific, Madrid, Spain).

The residual AIB measurement was performed in an ultimate 3000®UPLC (Thermo Fisher Scientific, Waltham, MA, USA) equipped with a UV–visible detector (Thermo Fisher Scientific, Waltham, MA, USA). The method was based on [75] but adapted to our conditions. Four hundred-microliter samples were derivatized with a mix of 12 µL diethyl ethoxymethylenemalonate (DEEMM) and 300 µL methanol. The reactions were carried out in screw-cap test tubes in an ultrasonic bath for 30 min, followed by heating at 80 °C for 2 h to degrade the excess DEEMM, and filtrated using 0.22-µm nylon syringe filters (Labbox Labware, Barcelona, Spain). The chromatographic analyses were conducted with an Accuore™ C18 column (Thermo Fisher Scientific, Waltham, MA). The applied triphasic gradient, which consists of phase A (methanol), phase B (acetonitrile), and phase C (25 mM acetate buffer, pH 6.7), is shown in Additional file 9: Table S4. To quantify the acetone production, 5 mL of the sample was mixed with 5 mL of 300 g/L NaCl. Then passed through a TRACE™ GC Ultra gas chromatograph (Thermo Fisher Scientific, Waltham, MA, USA) coupled with a flame ionization detector (FID), equipped with a 30 m × 0.25 mm × 0.25 µm HP-INNOWax capillary column coated with a layer of cross-linked polyethylene glycol (Agilent Technologies, Santa Clara, USA) at carrier gas helium flow rate of 1 mL/min. The oven temperature program was as follows: (1) 5 min at 50 °C, (2) temperature raised to 100 °C at the increasing rate of 1.5 °C/min. (3) then up to 215 °C at a rate of 3 °C/min and (4) was kept for 2 min more. The FID detector temperature was at 280 °C and the acetone was identified by its retention time. Quantification was made through an acetone calibration plot.

### DGD1 gene expression analysis by real-time qPCR

To determine whether *DGD1* expression is regulated by the gene *DGD2* and the amino acid AIB, strains BMV58 and IFO1802 and their *dgd2* mutants were cultured overnight in 5 mL of YNB liquid medium. The cells were shed once with a YNB medium without any nitrogen source. Then, they were inoculated into 50 mL YNB liquid medium in 250 mL flasks containing 1 mM AIB plus either 10 mM glutamine or 10 mM proline with an initial cell concentration of 1 × 10^6^ cells mL^−1^. YNB liquid medium containing either 10 mM glutamine or 10 mM proline as the sole nitrogen source was used as control media. Samples were taken when the cell concentration reached up to 1 × 10^7^ cells mL^−1^, frozen with liquid nitrogen, and stored at − 80 °C until mRNA extraction. Total RNA from the frozen samples was extracted using the QIAGEN RNA extraction kit. The total RNA samples were treated with 10 U of DNase I (Roche) and the cDNA was generated from 200 ng of total RNA using the NZY First-Strand cDNA synthesis kit (NZYTech, Lisbon, Portugal). The qPCRs were performed in a LightCycler® 480 Instrument (Roche, Mannheim, Germany) using the qPCR primers (Table 3). The expression of *DGD1* was normalized against the average expression of the reference genes *ACT1* and 18S rRNA. Then, each sample value was relativized against the average value of all samples and transformed into a log-2 value.

To determine the optimal AIB concentration for the expression experiment, the mutant strains *Su-DGD2Δ* and *Sk-DGD2Δ* were plated on YNB medium at the three different AIB concentrations assayed previously (1, 5, and 10 mM) plus either 10 mM glutamine or 10 mM proline at 25 °C for 3 days. The mutant strains Su-DGD1Δ and Sk-DGD1Δ and their parental strains AQ2901 and IFO1802 were used as control**.**

### Screening for orthologs in other yeast genomes

The sequences of three genes included in the cluster found in *Saccharomyces* (*DGD1*,* DGD2*, and AAP) were used to find orthologs in other yeast species. In total, 313 genome sequences of different species of budding yeasts [4] were used to run the genomic screening (Additional file 10: Table S5). A blastn [61] search was performed against the genomes with default parameters and word size set to 20. An in-house Python script was used to filter results by keeping hits with a total alignment length greater than 200 bp. Open-reading frames (ORFs) from the hits found were extracted and the longest ORFs for each gene were selected for further analysis. The translated sequences of the final selected ORFs were used to validate results by aligning against the Uniprot reference database (https://www.uniprot.org/) using Blastp tool. We also studied whether synteny was conserved when two of three genes of the cluster were found in the same yeast genome.

### Phylogeny reconstruction and comparison

Final validated *DGD1* and *DGD2* ORFs were used for phylogeny reconstruction. Sequences were translated into amino acids and aligned with MUSCLE [76] implemented in the MEGA v. 11.0.13 program [77].

The best evolutionary protein models based on empirical frequencies of amino acid replacements were selected with ProtTest 3.4.2 [78], and the best-fitting model for the Ddg1p alignment was the LG model [79] with gamma-distributed rates with an α shape parameter of 0.5623, and for the Dgd2p/Cha4p alignment, the JTT model [80] with a gamma distribution of rates with an α shape parameter of 0.8115, a proportion of invariable sites of 0.032 and the observed amino acid frequencies. Maximum likelihood phylogenies were obtained, with the appropriate model, by using the MEGA v. 11.0.13 program [77], with 500 bootstrap replicates.

Alternative phylogenetic hypotheses were compared with the Shimodaira-Hasegawa [41], the one-sided Kishino-Hasegawa [42], and the expected likelihood weights [43] tests implemented in Tree-Puzzle 5.3.rc [81].

## Supplementary Information

**Additional file 1: Table S1.** Source of the SaccharomycesAQ assemblies used for the blastn search. This table contains the source of the Saccharomyces genome sequences where the cluster containing the putative genes FT, AT, and AAP were present or not found.

**Additional file 2.**

**Additional file 3.**

**Additional file 4.**

**Additional file 5.**

**Additional file 6.**

**Additional file 7.**

**Additional file 8.**

**Additional file 9: Table S2.** Primers for AQ2901 and IFO1802 DGD1 alleles cloning used in this study. **Table S3.** Escherichia coli strains used in this study. **Table S4.** HPLC program for AIB quantification. **Table S6.** Primers for the DGD2 zinc finger motif sequencing.

**Additional file 10: Table S5.** Genomic screening of the gene cluster in budding yeast species. Green: orthologous gene present. Red: orthologous gene not found.

**Additional file 11.**

**Additional file 12.**

**Additional file 13.**

## Data Availability

Genome data used in this study are available in NCBI (Additional file 10: Table S5, see references therein). All data generated or analyzed during this study are included in this published article and its supplementary information files. For those results, based on three replicates, additional files containing the individual data values and their statistical analyses are provided, as indicated in the corresponding figure legends. All materials are also available upon request to the corresponding author.

## Abbreviations

AAP: Putative amino acid permease
*ACT1*: Actin gene
AIB: 2-Aminoisobutyric acid
*ALT1*: Alanine aminotransferase gene
AT: Aminotransferase-like protein
*DGD1*: Dialkylglycine decarboxylase gene
*DGD2*: AIB-responsive positive regulator gene
*GAP1*: General amino acid permease gene
HGT: Horizontal gene transfer
TF: Transcription factor
PLP: Pyridoxal phosphate
