# Supplementary figures and images for: A novel aminotransferase gene and its regulator acquired in Saccharomyces by a horizontal gene transfer event
Source: BMC Biol. 2023 May 8;21:102. doi: 10.1186/s12915-023-01566-6 (PMC10169451; doi:10.1186/s12915-023-01566-6)

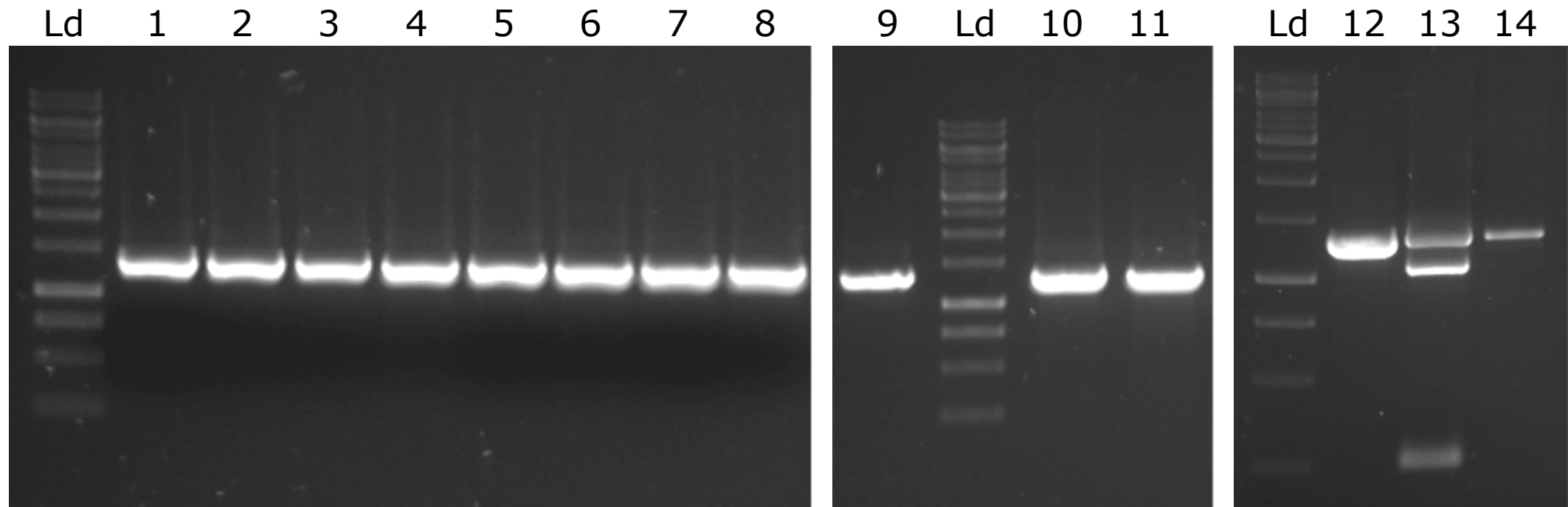

Supplement: Supplementary file 2 — Additional file 2. [file 12915_2023_1566_MOESM2_ESM.pdf]

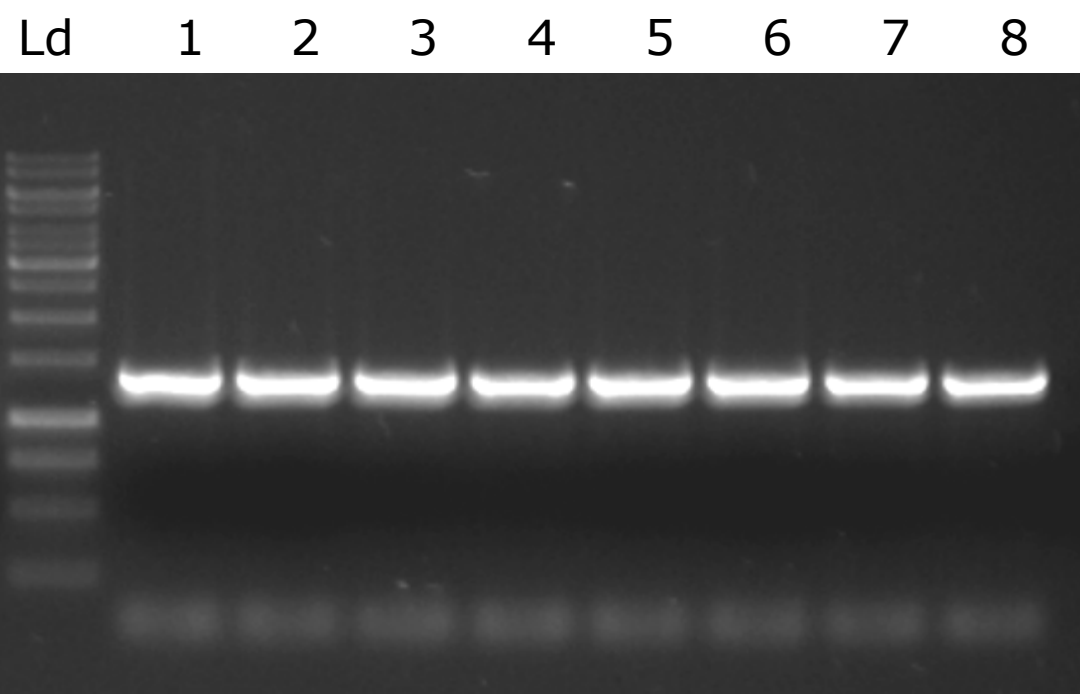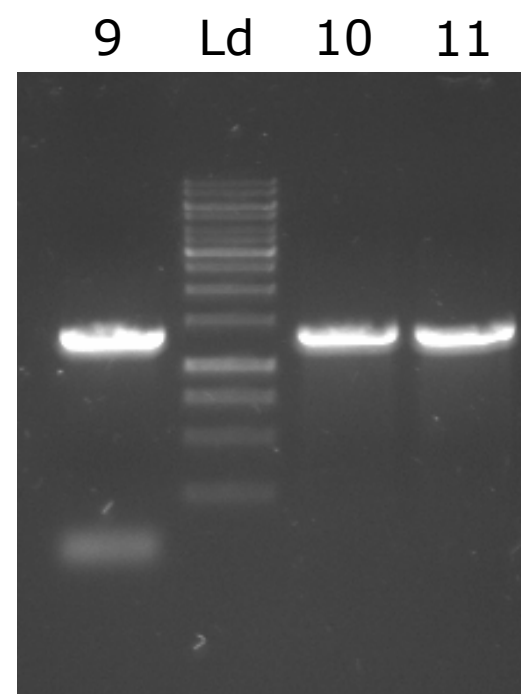

Supplement: Supplementary file 3 — Additional file 3. [file 12915_2023_1566_MOESM3_ESM.pdf]

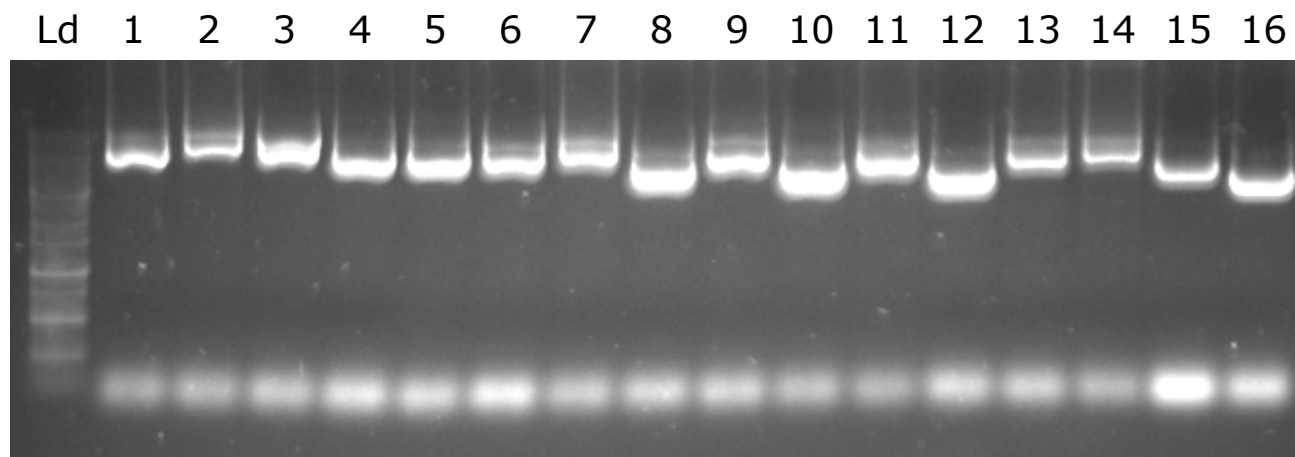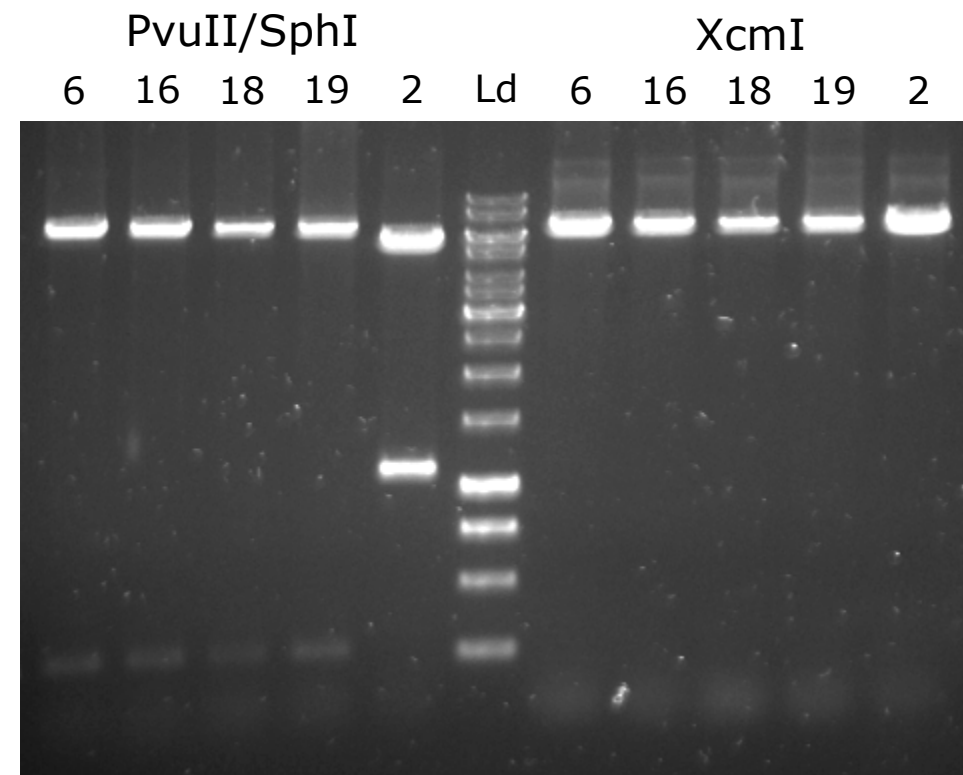

Supplement: Supplementary file 4 — Additional file 4. [file 12915_2023_1566_MOESM4_ESM.pdf]

Ld 1 2 3 Ld 4 5 6

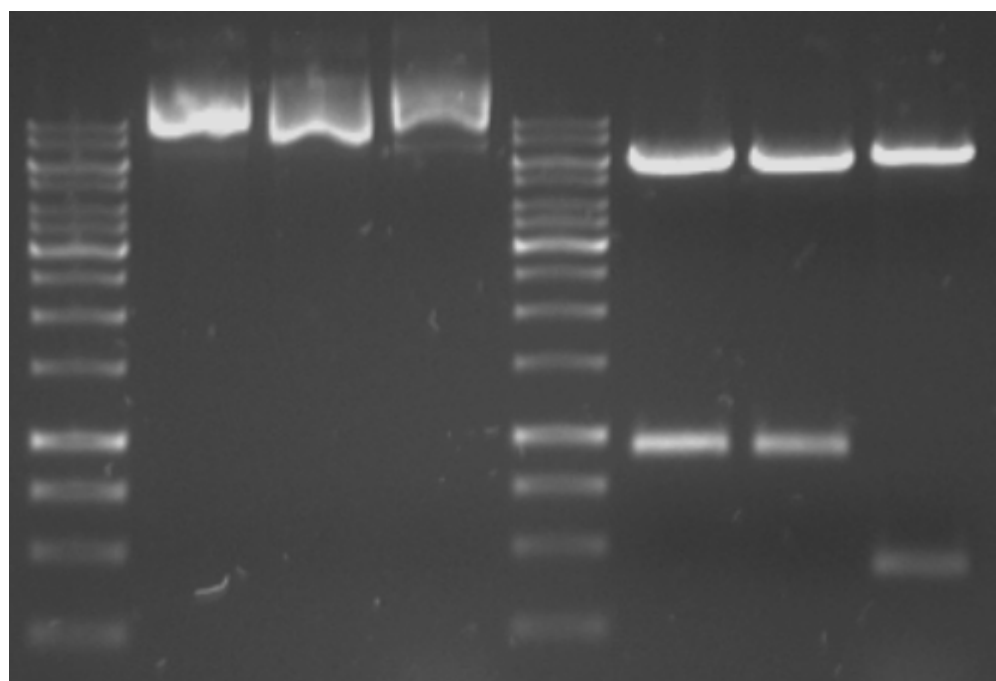

Supplement: Supplementary file 5 — Additional file 5. [file 12915_2023_1566_MOESM5_ESM.pdf]

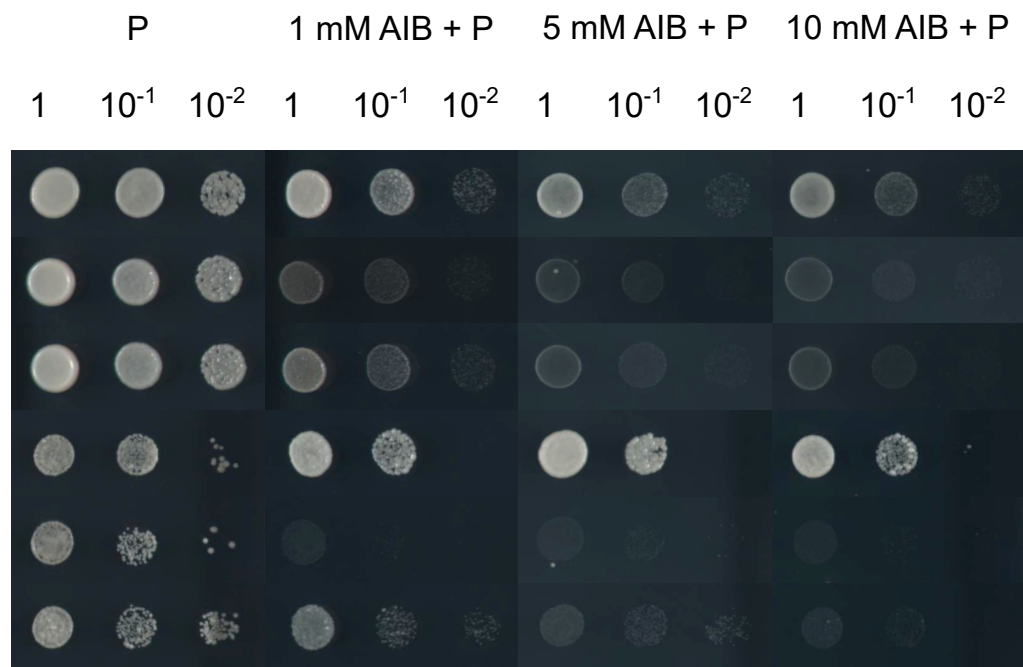

AQ2901

Su-DGD1 $\Delta$

Su-DGD2 $\Delta$

IFO1802

Sk-DGD1 $\Delta$

Sk-DGD2 $\Delta$

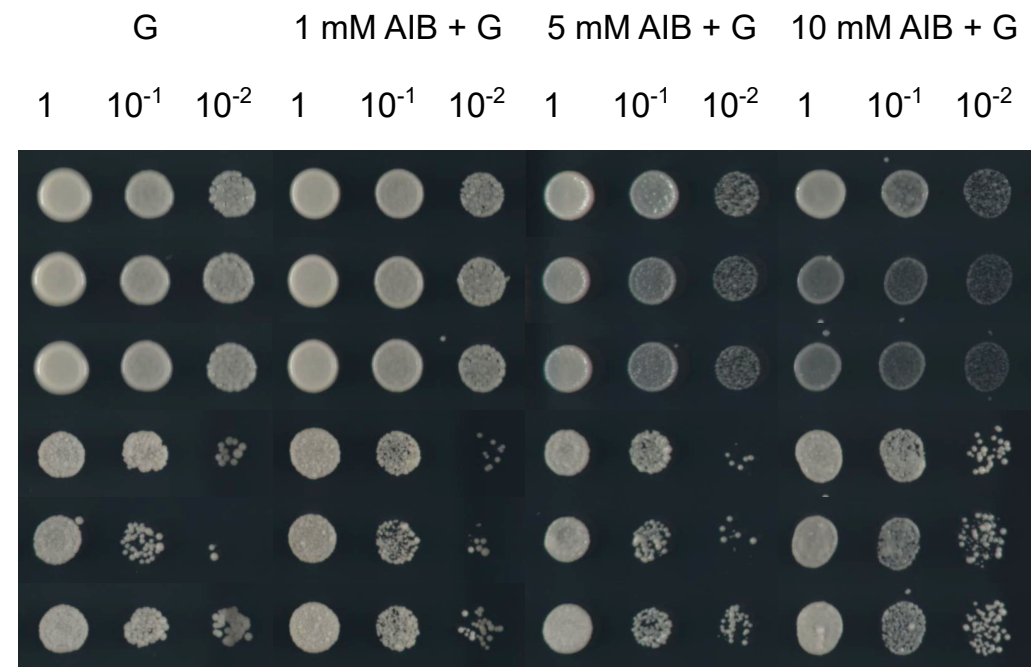

Supplement: Supplementary file 6 — Additional file 6. [file 12915_2023_1566_MOESM6_ESM.pdf]

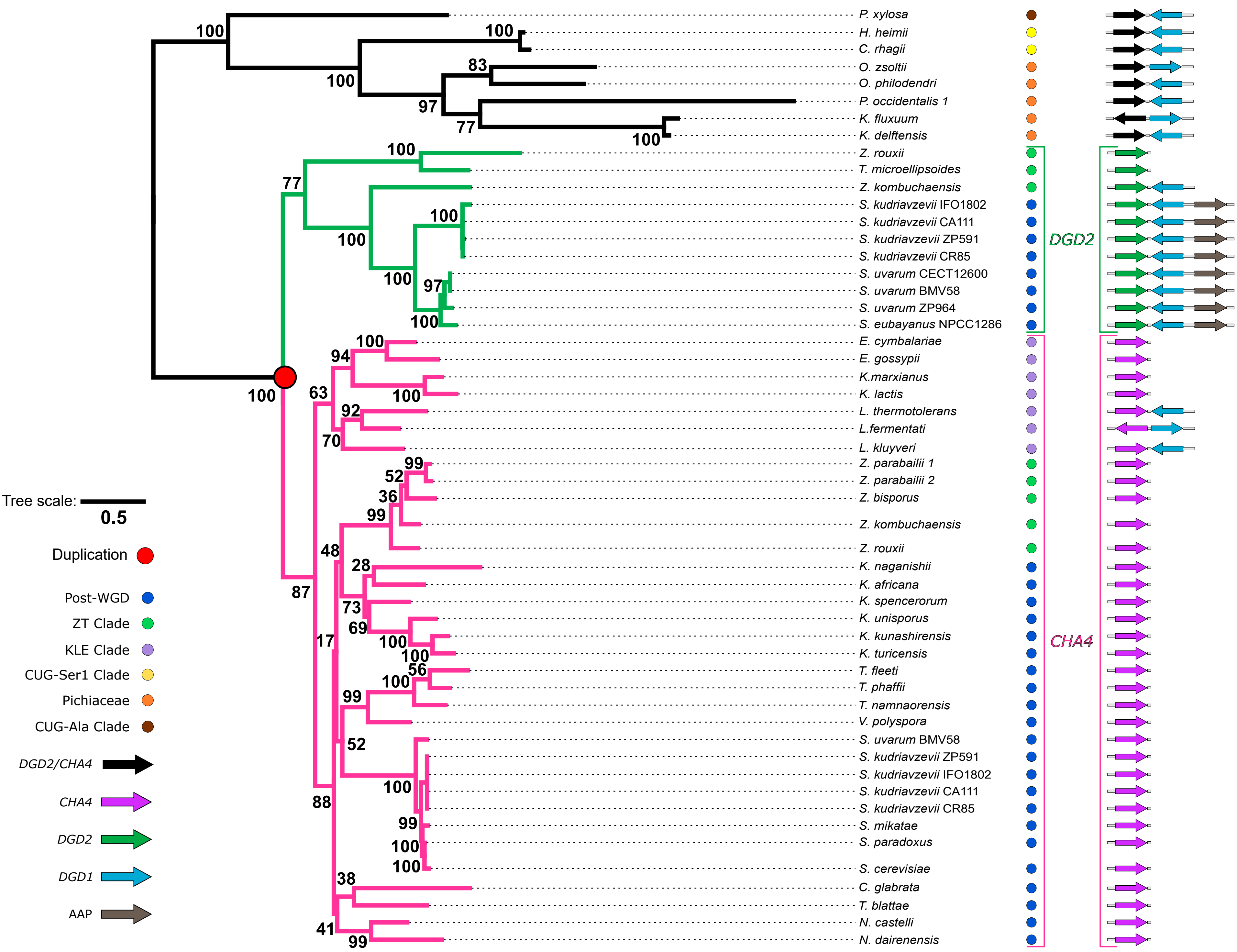

Supplement: Supplementary file 7 — Additional file 7. [file 12915_2023_1566_MOESM7_ESM.pdf]

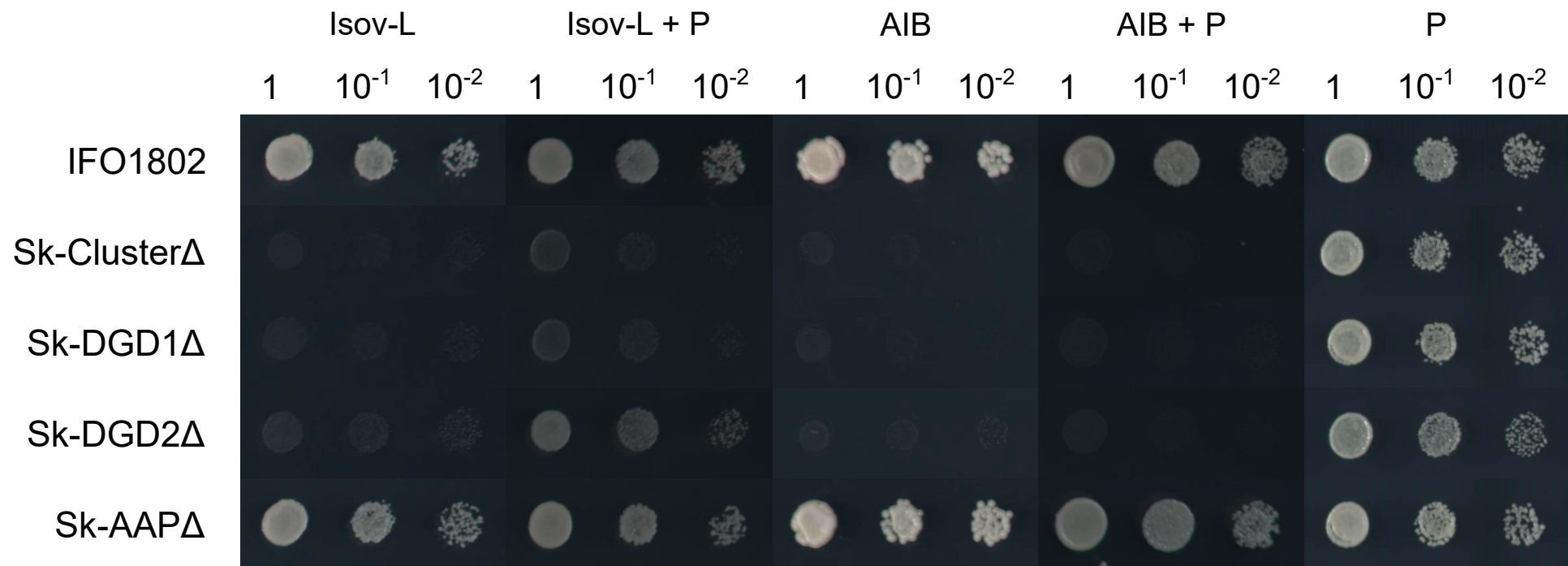

Supplement: Supplementary file 8 — Additional file 8. [file 12915_2023_1566_MOESM8_ESM.pdf]
