# Supplementary material for: A novel aminotransferase gene and its regulator acquired in Saccharomyces by a horizontal gene transfer event
Source: BMC Biol. 2023 May 8;21:102. doi: 10.1186/s12915-023-01566-6 (PMC10169451; doi:10.1186/s12915-023-01566-6)
Supplement: Supplementary file 9 — Additional file 9: Table S2. Primers for AQ2901 and IFO1802 DGD1 alleles cloning used in this study. Table S3. Escherichia coli strains used in this study. Table S4. HPLC program for AIB quantification. Table S6. Primers for the DGD2 zinc finger motif sequencing. [file 12915_2023_1566_MOESM9_ESM.pdf]

Supplementary Tables S2-S4, and S6

Table S2 Primers for AQ2901 and IFO1802 *DGD1* alleles cloning used in this study

**Cloning**	
Primer	Sequence (5' to 3')
Sk-DGD1-pYES-Fw	ACCATGACAAAACCATCC
Sk-DGD1-pYES-Rv	ACCCAATATCTCTTTAAAACTT
Su-DGD1-pYES-Fw	ACAAGTCACGTTTTTACTATG
Su-DGD1-pYES-Rv	GCCTAAAATTTCTTCCAAACT
**Sequencing**	
Primer	Sequence (5' to 3')
Sk-DGD1-Seq-Fw-1	ATGAGTTCAGGTGGTATGCT
Sk-DGD1-Seq-Fw-2	GCCATCAAGTTAGCCAAAGT
Sk-DGD1-Seq-Rv	AGCATACCACCTGAACTCAT
Su-DGD1-Seq-Fw	TTTGATTGTGGACGAAGCTC
Su-DGD1-Seq-Rv-1	GATACTCCTGTCATACCATG
Su-DGD1-Seq-Rv-2	GAGCTTCGTCCACAATCAAA
GAL1-Fw	AATATACCTCTATACTTTAACGTC
V5 C-term-Rv	ACCGAGGAGAGGGTTAGGGAT

Table S3 *Escherichia coli* strains used in this study

**Strain**	**Genotype**	**Reference**
TOP10F'	F´{*lac*I^{q} Tn*10* (Tet^{R})} *mcr*A Δ(*mrr-hsd*RMS-*mcr*BC) Φ80*lacZ*ΔM15 Δ*lac*X74 *rec*A1 *ara*D139 Δ(*ara-leu*)7697 *gal*U *gal*K *rps*L (Str^{R}) *end*A1 *nup*G	Invitrogen
TOP10F'-DGD1(Sk)	TOP10F', pYES-Sk-*DGD1*	This study
TOP10F'-DGD1(Su)	TOP10F', pYES-Su-*DGD1*	This study

Table S4 HPLC program for AIB quantification

**Time [min]**	**Flow [mL/min]**	**%A**	**%B**	**%C**
0.00	**Equilibration**			
0.00	0.4	2	8	90
0.00	**Run**			
0.00	0.4	2	8	90
2.00	0.4	2	8	90
4.00	0.4	2	5	93
6.52	0.4	2	8	90
17.00	0.4	2	48	50
23.50	0.4	2	8	90
25.50	**Stop Run**			

Table S6 Primers for the *DGD2* zinc finger motif sequencing

Primer	Sequence (5' to 3')
Sk-DGD2-Seq-Fw	GACACCCATACCCATTCGATAA
Sk-DGD2-Seq-Rv	CAAGACGGGTTTTTATAACGGAC
Su-DGD2-Seq-Fw	AACCACCCACACCCACATC
Su-DGD2-Seq-Rv	TCTGAAGACGATGGAACTGG

The primer sequences are specific for AQ2901 and IFO1802 backgrounds
